# Surface Engineering‐Induced d‐Band Center Down‐Regulation in High‐Entropy Alloy Nanowires for Enhanced Nanozyme Catalysis

**DOI:** 10.1002/advs.202502354

**Published:** 2025-04-07

**Authors:** Kunyang Feng, Hanting Wang, Song Zhou, Wei Zhang, Chonghai Gong, Yuxin He, Yusen Wang, Wenchong Dai, Jianbo Li, Zhengwei Zhang, Siqiao Li

**Affiliations:** ^1^ School of Basic Medical Sciences Chongqing Medical University Chongqing 400016 China; ^2^ Chongqing Key Laboratory of Forensic Medicine Chongqing Medical University Chongqing 400016 China; ^3^ College of Pharmacy Chongqing Medical University Chongqing 400016 China; ^4^ School of Management Shanxi Medical University Shanxi 030001 China

**Keywords:** DFT calculations, high‐entropy alloy, nanozyme, photothermal effect, surface modification

## Abstract

High‐entropy alloys (HEAs) have garnered extensive attention owing to their broad compositional tunability and high catalytic activity. However, precisely modulating the enzyme‐like activity of HEAs and enhancing their biocompatibility for biological applications remain severely challenging. Herein, PtRuFeCoNi HEA nanowires (NWs) are synthesized by adjusting the metal composition and surface‐engineered with polydopamine (PDA) to form HEA NWs@PDA nanozymes (HEzymes@PDA) with superior catalytic activity and photothermal properties. Density functional theory calculations and the Sabatier principle reveal that self‐polymerized PDA surface engineering moderately lowers the d‐band center of the HEAs, optimizes the surface charge distribution, and enhances the adsorption–desorption efficiency of the substrates. As a proof‐of‐concept, the HEzymes@PDA are synergistically integrated with hydrogels for biosensing analysis. This study presents an innovative paradigm for designing highly active HEA nanozymes via surface engineering and demonstrates their immense potential in catalytic sensing applications.

## Graphical abstract

PtRuFeCoNi HEA NWs are synthesized by adjusting the metal composition and surface‐engineered with PDA to create HEA NWs@PDA nanozymes with superior catalytic activities and photothermal properties. DFT calculations confirm that self‐polymerized PDA surface engineering moderately lowers the d‐band center of the HEAs, optimizes the surface charge distribution, and enhances the adsorption–desorption efficiency of the substrates.

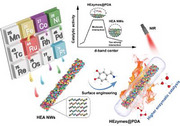

## 1 Introduction

Nanozymes are a class of enzyme mimetics that integrate the distinctive characteristics of nanomaterials with their catalytic functionalities.^[^ ^1^ ^]^ They can emulate the functions of natural enzymes, including peroxidase (POD) and oxidase (OXD), and offer exclusive advantages such as low cost, high stability, ease of modification, adjustable catalytic activity, and scalability for large‐scale production.^[^ ^2^ ^]^ The distinct physicochemical properties of nanomaterials, including their size, morphology, and active sites, provide exceptional opportunities to fine‐tune their catalytic performance, applying in biochemical sensing, disease diagnosis and treatment, and environmental protection.^[^ ^3^, ^4^ ^]^ Various nanocatalyst materials, including metal‐based, carbon‐based, and metal oxide/sulfide nanozymes, have been developed.^[^ ^5^ ^]^ However, challenges such as limited catalytic activity, complex catalytic mechanisms, and poor biocompatibility persist, necessitating the development of innovative nanozymes, advanced activity modulation strategies, and deeper investigations into their catalytic mechanisms.^[^ ^6^ ^]^

High‐entropy alloys (HEAs) are single‐phase solid‐solution alloys with a simple crystal structure that are formed from five or more metals in equal or approximately equal proportions, each contributing 5–35% of the atomic ratio.^[^ ^7^ ^]^ Compared with conventional alloy materials, HEAs exhibit excellent hardness, wear resistance, and thermal stability, making them appealing in numerous fields such as chemical engineering, energy, and thermoelectricity.^[^ ^8^, ^9^ ^]^ Moreover, their intrinsically high entropy eliminates immiscibility boundaries between elements, thereby facilitating robust control of their composition and optimizing catalytic performance.^[^ ^10^ ^]^ Entropy‐mediated strategies enable the precise design of HEAs by controlling the lattice distortion and elemental composition through tailored preparation condition. This approach allows the creation of active sites with specific geometric structures and electronic characteristics, offering significant potential for enhanced catalytic performance and diverse applications.^[^ ^11^, ^12^ ^]^ The catalytic activity and dispersion of HEAs depend on the types and proportions of their constituent metals.^[^ ^13^ ^]^ Variations in atomic sizes of the elements in the HEAs introduce substantial lattice distortions, thereby increasing the energy barrier for atomic diffusion and enhancing their stability and formation.^[^ ^14^ ^]^ In addition, the cocktail effect in the HEAs modulates the adsorption of reactants and intermediates at active sites through synergistic interactions among the elements.^[^ ^15^ ^]^ Density functional theory (DFT) calculations have been extensively employed to elucidate the mechanisms underlying the catalytic activity of HEAs, focusing on their geometric and electronic structures, as well as substrate adsorption and reaction processes.^[^ ^16^ ^]^ Although HEAs show promise for various catalytic reactions, their complex structures hinder precise regulation, potentially resulting in a suboptimal enzyme‐like activity. Moreover, their low biocompatibility and potential toxicity limit their biomedical applications. Notably, the introduction of surface modification engineering to HEAs offers a promising solution to these problems.^[^ ^17^ ^]^ By functionalizing small molecules, macromolecules, and polymers, HEAs can optimize their catalytic potential and biocompatibility, advancing the development of high‐entropy nanozymes (HEzymes) and the field of nanozymology.

The catalytic performance of nanozymes is closely linked to their surface structural properties and electronic distributions.^[^ ^18^ ^]^ Surface modification engineering and ligand alterations are effective strategies for optimizing catalytic efficiency by exposing or increasing the number of active sites, modifying surface properties, and enhancing stability and biocompatibility.^[^ ^19^ ^]^ Polydopamine (PDA) coating, inspired by the adhesive properties of mussel foot proteins, has emerged as a multifunctional surface modification approach.^[^ ^20^ ^]^ PDA surfaces are rich in catechol, imine, and amine groups, which enable strong metal‐ion chelation, hydrophilicity, biocompatibility, and adhesion.^[^ ^21^ ^]^ PDA coatings alter the surface electronic and chemical properties of materials, and their strong near‐infrared (NIR) absorption can impart photothermal conversion efficiency under 808 nm irradiation.^[^ ^22^ ^]^ When applied to noble metals, PDA coatings generally exhibit enhanced nanozyme activity through induced accumulation and electron transfer effects.^[^ ^23^ ^]^ The dual amplification of the catalytic performance of HEzymes can be accomplished through a combined strategy of structural design and surface modification. Integrating PDA with HEzymes for dual‐function regulation of the enzymatic activity and photothermal effects remains an unexplored avenue. According to the Sabatier principle, optimal catalytic performance requires appropriate interactions between catalysts and reactants. In addition, the position of the d‐band center in HEzymes plays a critical role in determining adsorption strength, thereby influencing the catalytic performance.^[^ ^24^ ^]^ Therefore, the Sabatier principle and d‐band center theory can provide theoretical guidance for evaluating surface modification strategies and designing functionalized HEzymes.

In this study, we synthesized PtRuFeCoNi HEA nanowires (NWs) by adjusting the metal composition and subsequently surface‐engineered them with PDA to obtain HEA NWs@PDA nanozymes (HEzymes@PDA) with high POD‐like activities. HEzymes@PDA exhibited superior catalytic activity and dispersibility compared with HEA NWs. The photothermal effect stimulated by the PDA coating significantly increased the local microenvironment temperature of HEzymes@PDA under NIR irradiation, thereby enhancing the catalytic efficiency. DFT calculations were employed to explore the electronic structure and catalytic mechanisms of the HEA NWs, revealing the impact of the d‐orbital hybridization between Pt, Ru, and transition metals on electron transfer and substrate adsorption. According to the Sabatier principle, the enhanced catalytic performance of HEzymes@PDA resulted from the moderate adjustment of the surface charge distribution and a slight reduction in the d‐band center, which optimized the adsorption–desorption efficiency of the substrates. To validate the theoretical findings, a colorimetric sensing platform based on HEzymes@PDA was developed for practical applications in detecting acetylcholinesterase (AChE) activity and methomyl in fruits. This study demonstrates that surface modification engineering enhances the catalytic performance of HEzymes and provides insights into the relationship between the Sabatier principle and d‐band center for improving nanozyme catalysis, offering new perspectives for HEzyme design and biosensing applications.

## 2 Results and Discussion

### 2.1 Characterization of PtRuFeCoNi HEzymes@PDA

**Figure 1:**
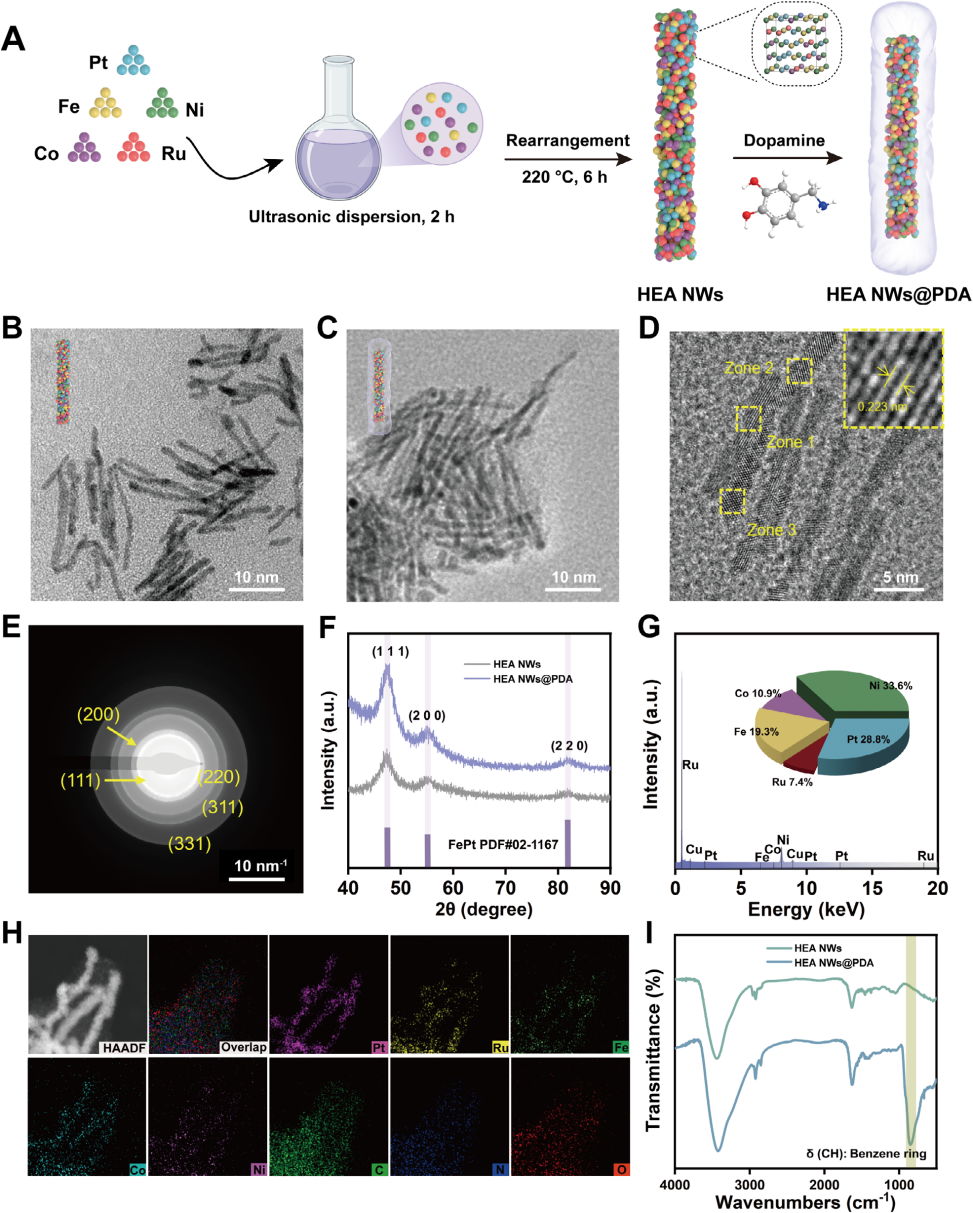
Synthesis and characterizations of PtRuFeCoNi HEA NWs@PDA. A) Schematic illustration showing the formation process of HEA NWs@PDA. B) TEM image of HEA NWs. C) TEM image of HEA NWs@PDA. D) HRTEM image of HEA NWs. The inset diagram shows the atomic arrangement. E) SAED image of HEA NWs. F) XRD pattern of HEA NWs and HEA NWs@PDA. G) EDS spectrum of HEA NWs and metallic element contents obtained by ICP‐OES. H) HAADF‐STEM and corresponding elemental mapping images of HEA NWs@PDA. I) FT‐IR spectra of HEA NWs and HEA NWs@PDA.

PtRuFeCoNi HEA NWs were synthesized using a simple low‐temperature oil‐phase method, and HEA NWs@PDA were prepared using a self‐polymerized dopamine strategy applied to the HEA NWs (**Figure** **1**A).^[^ ^12^, ^15^, ^25^ ^]^ First, platinum (II) acetylacetonate (Pt(acac)_2_), ruthenium (III) acetylacetonate (Ru(acac)_3_), iron (III) acetylacetonate (Fe(acac)_3_), cobalt (III) acetylacetonate (Co(acac)_3_), and nickel (II) acetylacetonate (Ni(acac)_2_) were used as metal precursors. Oleylamine (OAm) acted as the solvent, cetyltrimethylammonium bromide (CTAB) served as the structure‐directing agent, and molybdenum hexacarbonyl (Mo(CO)_6_) and glucose were used as reducing agents. After obtaining the PtRuFeCoNi HEA NWs, they were dispersed in anhydrous ethanol and reacted with dopamine in a Tris‐HCl solution. Oxidized dopamine aggregated to form PDA on the surfaces of the HEA NWs, primarily due to the rich presence of catechol, imine, and amine groups in PDA, which conferred an excellent metal‐chelating ability.

The transmission electron microscopy (TEM) image showed that the HEA NWs possessed a uniform wire morphology, with an average length of 14.41 ± 1.43 nm and width of 1.85 ± 0.34 nm (Figure 1B). The self‐polymerized PDA successfully coated the surface of the HEA NWs, with the HEA NWs@PDA retaining its original filamentary structure (Figure 1C). The high‐resolution TEM (HRTEM) image of the HEA NWs displayed an ordered fringe pattern, with an average interplanar spacing of ≈0.223 nm, which corresponded to the (1 1 1) crystal face (Figure 1D). Meanwhile, the integrated intensity of the (1 1 1) lattice in the chosen area revealed that the average spacing between lattices, determined through fast Fourier transform (FFT) analysis, ranged from 0.223 (zone 1) to 0.224 nm (zone 2), and then to 0.225 nm (zone 3), demonstrating lattice distortion in the synthesized HEA NWs (Figure S1, Supporting Information).^[^ ^17^, ^26^ ^]^ In addition, the selected area electron diffraction (SAED) image clearly showed that the HEA NWs had five crystal planes, namely (1 1 1), (2 0 0), (2 2 0), (3 1 1), and (3 3 1), verifying the face‐centered cubic (FCC) structure of the synthesized HEA NWs (Figure 1E).^[^ ^27^ ^]^ The three characteristic peaks in the X‐ray diffraction (XRD) pattern of the HEA NWs corresponded to the FCC structure of FePt (PDF#02‐1167), whereas the diffraction peak intensity of HEA NWs@PDA exhibited a notable increase compared with that of the HEA NWs, which was likely due to the PDA coating (Figure 1F).^[^ ^28^ ^]^ The positions of the broad diffraction peaks were significantly shifted compared with those of pure Pt, Ru, Fe, Co, and Ni, proving the incorporation of these metals into the HEA NWs structure to form an FCC structure (Figure S2, Supporting Information).^[^ ^16b^ ^]^ This data provided initial evidence that the synthesis of HEA NWs and surface modification with PDA were successful.

High‐angle annular dark‐field scanning TEM (HAADF‐STEM) and the corresponding elemental mapping images showed that the five elements (Pt, Ru, Fe, Co, and Ni) were uniformly distributed throughout the entire NW (Figure S3, Supporting Information). In addition, the energy‐dispersive X‐ray spectroscopy (EDS) spectrum of the HEA NWs further confirmed the coexistence of Pt, Ru, Fe, Co, and Ni (Figure 1G), demonstrating the successful fabrication of PtRuFeCoNi HEA NWs. The inductively coupled plasma optical emission spectrometry (ICP‐OES) results showed that the relative atomic ratios of Pt, Ru, Fe, Co, and Ni were 29:7:19:11:34 (inset of Figure 1G). Based on this calculation, the configurational entropy of the HEA NWs was determined to be 1.83 R, which aligned with the high‐entropy level of the HEA NWs, classifying the material as an HEA.^[^ ^8a^ ^]^ The EDS mapping images of HEA NWs@PDA clearly showed that, in addition to the distribution of the five metals, the NW coating also contained abundant C, N, and O, thereby verifying the encapsulation of PDA (Figure 1H). Fourier‐transform infrared spectroscopy (FT‐IR) was employed to analyze the changes in the functional groups on the surface of the HEA NWs after PDA encapsulation. The diffraction peak intensity of HEA NWs@PDA was higher than that of the HEA NWs, and out‐of‐plane distortion of the C–H bond of the aromatic ring emerged, confirming the successful modification with PDA (Figure 1I).^[^ ^29^ ^]^ X‐ray photoelectron spectroscopy (XPS) was used to evaluate the detailed surface elemental composition of the as‐prepared HEA NWs and HEA NWs@PDA. The Pt 4f, Ru 3p, Fe 2p, Co 2p, and Ni 2p binding energy peaks confirmed the successful formation of HEA NWs (Figure S4A, Supporting Information). The peaks of Pt 4f_7/2_ and Pt 4f_5/2_ were located at 71.12 and 74.46 eV, respectively, while those of Ru 3p_3/2_ and Ru 3p_1/2_ corresponded to 463.15 and 485.02 eV (Figure S4B,C, Supporting Information).^[^ ^12^ ^]^ The peaks of Fe 2p_3/2_ and Fe 2p_1/2_ were located at 712.09 and 724.45 eV, respectively, whereas those of Co 2p_3/2_ and Co 2p_1/2_ corresponded to 780.91 and 796.79 eV (Figure S4D,E, Supporting Information). The two peaks at 855.75 and 873.45 eV originated from Ni 2p_3/2_ and Ni 2p_1/2_ electrons, respectively (Figure S4F, Supporting Information).^[^ ^15^, ^30^ ^]^ These results indicated that the HEA NWs contained five metals, namely Pt, Ru, Fe, Co, and Ni, and presented diverse valence states. However, the XPS results of the HEA NWs@PDA displayed a substantial reduction in the peak signals of each metal element, while the signal of C increased, indicating that PDA successfully coated the surface of the HEA NWs (Figures S4 and S5, Supporting Information). HEA NWs exhibited a positive charge of 7.94 mV, whereas HEA NWs@PDA had a negative charge of ≈ −16.52 mV, further confirming the successful modification with PDA (Figure S6, Supporting Information). Using the same method, we successfully synthesized FeCoNi and RuFeCoNi alloy nanoparticles (NPs) with fewer metal precursors. The TEM images of FeCoNi NPs and RuFeCoNi NPs showed monodisperse spherical morphologies with average diameters of 1.51 ± 0.30 and 2.89 ± 0.29 nm, respectively (Figures S7A,B and S8A,B, Supporting Information). The elemental ratios of the FeCoNi and RuFeCoNi NPs were also determined by ICP‐OES (Figures S7C and S8C, Supporting Information).

### 2.2 Catalytic Activity of POD‐Like Enzymes in HEzymes@PDA

**Figure 2:**
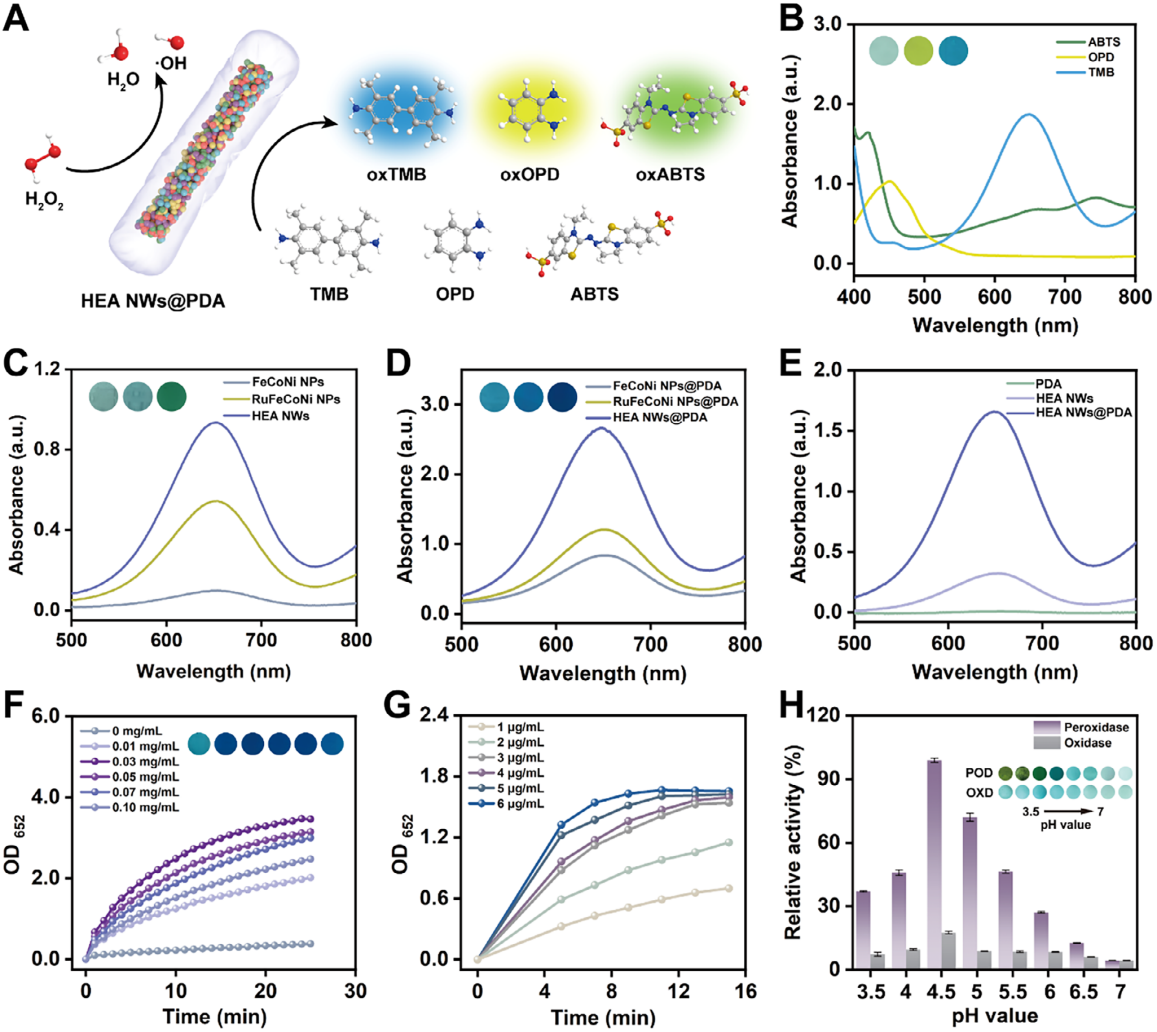
POD‐like activity and optimization of HEA NWs and HEA NWs@PDA. A) Schematic diagram of the POD‐like activity evaluations of HEA NWs@PDA with TMB, OPD, and ABTS. B) UV–vis absorption spectra and visual colors of different chromogenic reactions of HEA NWs@PDA. C) UV–vis absorption spectra and visual colors of TMB chromogenic systems in the presence of FeCoNi NPs, RuFeCoNi NPs, and HEA NWs. D) UV–vis absorption spectra and visual colors of TMB chromogenic systems in the presence of FeCoNi NPs@PDA, RuFeCoNi NPs@PDA, and HEA NWs@PDA. E) UV–vis absorption spectra of TMB chromogenic systems in the presence of PDA, HEA NWs, and HEA NWs@PDA. F) Time‐dependent POD‐like activities of HEA NWs@PDA obtained by different dopamine concentrations. G) Time‐dependent POD‐like activities of different concentrations of HEA NWs@PDA. H) OXD‐like and POD‐like activities and visual colors of HEA NWs@PDA at different pH values. Data are presented as the mean ± SD (standard deviation) (n = 6 independent samples).

We initially validated the enzymatic catalytic performance of HEA NWs and HEA NWs@PDA by using 3,3′,5,5′‐tetramethylbenzidine (TMB), 2,2′‐azino‐bis (3‐ethylbenzothiazoline‐6‐sulfonic acid) diammonium salt (ABTS), and o‐phenylenediamine (OPD) as chromogenic substrates. In the presence of H_2_O_2_, the classical peaks of the colored products (oxTMB: 652 nm, oxABTS: 415 nm, and oxOPD: 431 nm) were monitored using UV–vis spectroscopy (**Figure** **2**A,B; Figure S9, Supporting Information). The results confirmed that the HEA NWs and HEA NWs@PDA possessed good substrate universality. Compared with the POD‐like activities of FeCoNi NPs and RuFeCoNi NPs, the HEA NWs exhibited more potent enzymatic activity (Figure 2C; Figure S10, Supporting Information). The improvement in the enzymatic activity of the RuFeCoNi NPs over that of the FeCoNi NPs was attributed to doping with precious metals. The exceptional activity of the HEA NWs was likely due to the shift in the d‐band center caused by lattice distortion, which enhanced the adsorption capacity of the HEzymes. In addition, the synergy between double precious and transition metals improved the catalytic activity.^[^ ^13^, ^31^ ^]^ To validate the universality of PDA modification, PDA coatings were successfully deposited on the surfaces of FeCoNi NPs, RuFeCoNi NPs, and HEA NWs. Compared with alloy materials without surface modification, the catalytic performance of PDA‐coated materials was significantly enhanced, with HEA NWs@PDA displaying the highest enzymatic activity (Figure 2D; Figures S11‒S13, Supporting Information). PDA modification enhanced the dispersibility of alloy materials, enabling their active sites to more readily bind with substrate, thereby improving the catalytic performance.^[^ ^32^ ^]^ Furthermore, due to the exceptional hydrophilicity and biocompatibility of PDA, the solubility and dispersion of HEA NWs@PDA were markedly improved in comparison with those of HEA NWs (Figure S14, Supporting Information), further elevating their enzymatic activity and making them more suitable for biological applications.

To exclude the influence of PDA, we synthesized pure PDA without HEA NWs and investigated its POD‐like activity. The results indicated that pure PDA lacked catalytic capability, and HEA NWs@PDA achieved a “1 + 1 > 2” effect in the catalytic activity compared with HEA NWs and PDA alone (Figure 2E). By varying the dopamine concentration during the formation of HEA NWs@PDA, the obtained samples exhibited diverse POD‐like activities. When a higher dopamine concentration was used, the enzyme‐like activity decreased (Figure 2F), likely due to the formation of overly thick PDA coatings that masked the active sites of the HEA NWs, thereby influencing their catalytic performance.^[^ ^33^ ^]^ Thus, the optimal concentration of the PDA coating was selected to be 0.03 mg mL^−1^. To further enhance the enzyme‐like activity of HEA NWs@PDA, catalytic parameters such as concentration and pH were optimized. The absorbance of the reaction system was highly sensitive to the HEA NWs@PDA concentration and gradually increased with increasing material concentration (Figure 2G). Based on the equilibrium of the colorimetric reaction, the appropriate concentration of HEA NWs@PDA was selected as 5 µg mL^−1^. In different pH systems, HEA NWs@PDA exhibited the strongest enzyme‐like activity at pH 4.5 (Figure 2H). Furthermore, the POD‐ and OXD‐like activities of HEzymes@PDA were systematically examined, revealing that the expression of the OXD‐like activity was extremely limited compared with that of the POD‐like activity (Figure 2H). Therefore, the influence of the OXD‐like activity was considered negligible.

### 2.3 Impact of the Photothermal Effect on the POD‐Like Activity of HEzymes@PDA

**Figure 3:**
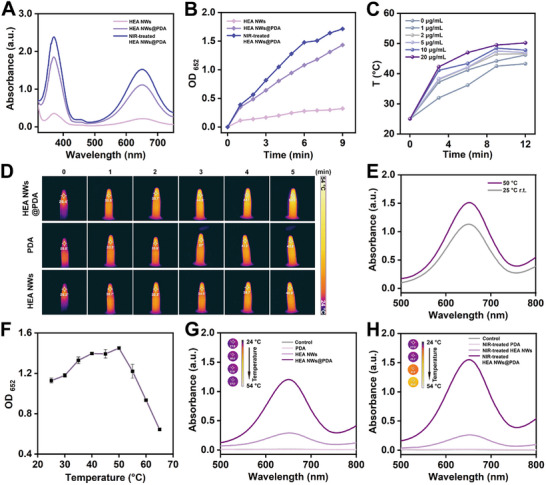
The photothermal effect on the POD‐like activity and mechanism of HEA NWs@PDA. A) UV–vis absorption spectra of TMB chromogenic systems in the presence of HEA NWs, HEA NWs@PDA, and NIR‐treated HEA NWs@PDA. B) Time‐dependent POD‐like activities of HEA NWs, HEA NWs@PDA, and NIR‐treated HEA NWs@PDA. C) Photothermal performance at different concentrations of HEA NWs@PDA. D) Temperature‐time thermograms of infrared thermal images of PDA, HEA NWs, and HEA NWs@PDA under NIR light for 0‒5 min. E) UV–vis absorption spectra of TMB chromogenic systems in the presence of HEA NWs@PDA at 50 °C and room temperature under dark field. F) The effect of different temperatures on the POD‐like activity of HEA NWs@PDA. Data are presented as the mean ± SD (standard deviation) (n = 6 independent samples). G) UV–vis absorption spectra of TMB chromogenic systems in the absence and presence of PDA, HEA NWs, and HEA NWs@PDA under dark field. The inset shows the above systems infrared thermal images (control: 27.6 °C, PDA: 28.2 °C, HEA NWs: 27.8 °C, HEA NWs@PDA: 27.2 °C). H) UV–vis absorption spectra of TMB chromogenic systems in the absence and presence of NIR‐treated PDA, NIR‐treated HEA NWs, and NIR‐treated HEA NWs@PDA. The inset shows the above systems infrared thermal images (control: 27.8 °C, PDA: 28.2 °C, HEA NWs: 31.9 °C, HEA NWs@PDA: 39.2 °C).

Owing to its molecular structure and physical properties, PDA as a coating not only enhanced the POD‐like activity of HEA NWs but also modulates their photothermal properties.^[^ ^34^ ^]^ Inspired by the temperature‐dependent mechanism of H_2_O_2_ decomposition, we investigated the effect of the photothermal mechanism on the POD‐like activity of HEA NWs@PDA. **Figure** **3**A shows that after PDA modification and NIR treatment, the catalytic activity of the HEA NWs steadily increased. As the colorimetric reaction time extended, the absorbance of HEA NWs and HEA NWs@PDA continuously increased, with HEA NWs@PDA showing the most significant enhancement under NIR irradiation (Figure 3B), indicating that NIR‐treated HEA NWs@PDA possessed a superior POD‐like activity. Figure 3C shows the temperature variation curves for HEA NWs@PDA at various concentrations over time, indicating a distinct concentration‐dependent temperature elevation. The irradiation power of the laser was carefully optimized to achieve the best photothermal performance, with 1.2 W cm^−1^ chosen as the optimal power (Figure S15, Supporting Information). In addition, HEA NWs@PDA exhibited exceptional photothermal stability, with no apparent temperature change after four on/off laser irradiation cycles (Figure S16, Supporting Information).

To clarify the underlying mechanism by which NIR irradiation enhances the catalytic activity, the photothermal effects of PDA, HEA NWs, and HEA NWs@PDA were compared, and temperature variations were recorded using a thermal imager. The results indicated that both PDA and HEA NWs possessed certain photothermal conversion capabilities, while HEA NWs@PDA displayed the optimal photothermal conversion performance (Figure 3D). This confirmed that PDA modification enhanced the photothermal properties of HEA NWs, enabling a synergistic effect. To further validate this, a water bath heating method was applied as an alternative to NIR processing. When treated at 50 °C in the water bath, the absorbance increase observed was similar to that from the NIR treatment, aligning with the temperature optimization results (Figure 3A,E). The temperature optimization results also demonstrated that the optimal temperature for HEA NWs@PDA to exert catalytic activity was 50 °C (Figure 3F). These findings suggested that the enhancement of the POD‐like activity of HEA NWs@PDA following NIR irradiation was primarily due to the elevation of the system temperature.^[^ ^35^ ^]^ To analyze the impact of PDA on the photothermal performance, the catalytic activities of HEA NWs modified with different amounts of PDA were tested under both dark‐field and NIR light conditions. The PDA modification of HEA NWs significantly enhanced the POD activity, and NIR light irradiation could further improve the catalytic performance (Figure S17, Supporting Information). Furthermore, absorbance and temperature changes in the chromogenic systems of PDA, HEA NWs, and HEA NWs@PDA were compared under dark‐field and NIR irradiation (Figure 3G,H). The results showed that the temperature change trends for each system were consistent under dark‐field conditions, whereas the catalytic system containing HEA NWs@PDA exhibited a significant temperature change and relative increase in absorbance under NIR irradiation.^[^ ^36^ ^]^ Under NIR irradiation, the localized temperature of the HEA NWs@PDA microenvironment increased, promoting the decomposition of H_2_O_2_ and increasing the diffusion rate of free radicals, thereby further enhancing the catalytic activity. Consequently, we initially designed HEA NWs with POD‐like activity, which was subsequently improved through PDA coating to enhance their enzyme‐like activity and biocompatibility. The catalytic activity was further augmented by leveraging the synergistic photothermal effect of PDA under NIR irradiation (Figure S18, Supporting Information). Finally, the stabilities of HEA NWs and HEA NWs@PDA were investigated (Figure S19, Supporting Information). After being exposed to various environmental conditions for 7 d, as well as stored at room temperature for 30 d, no significant alterations were observed in the catalytic or photothermal performance of HEA NWs@PDA. These results demonstrated that HEzymes have excellent enzyme activity stability.

### 2.4 Kinetic Determination of HEzymes@PDA and Free‐Radical Analysis

**Figure 4:**
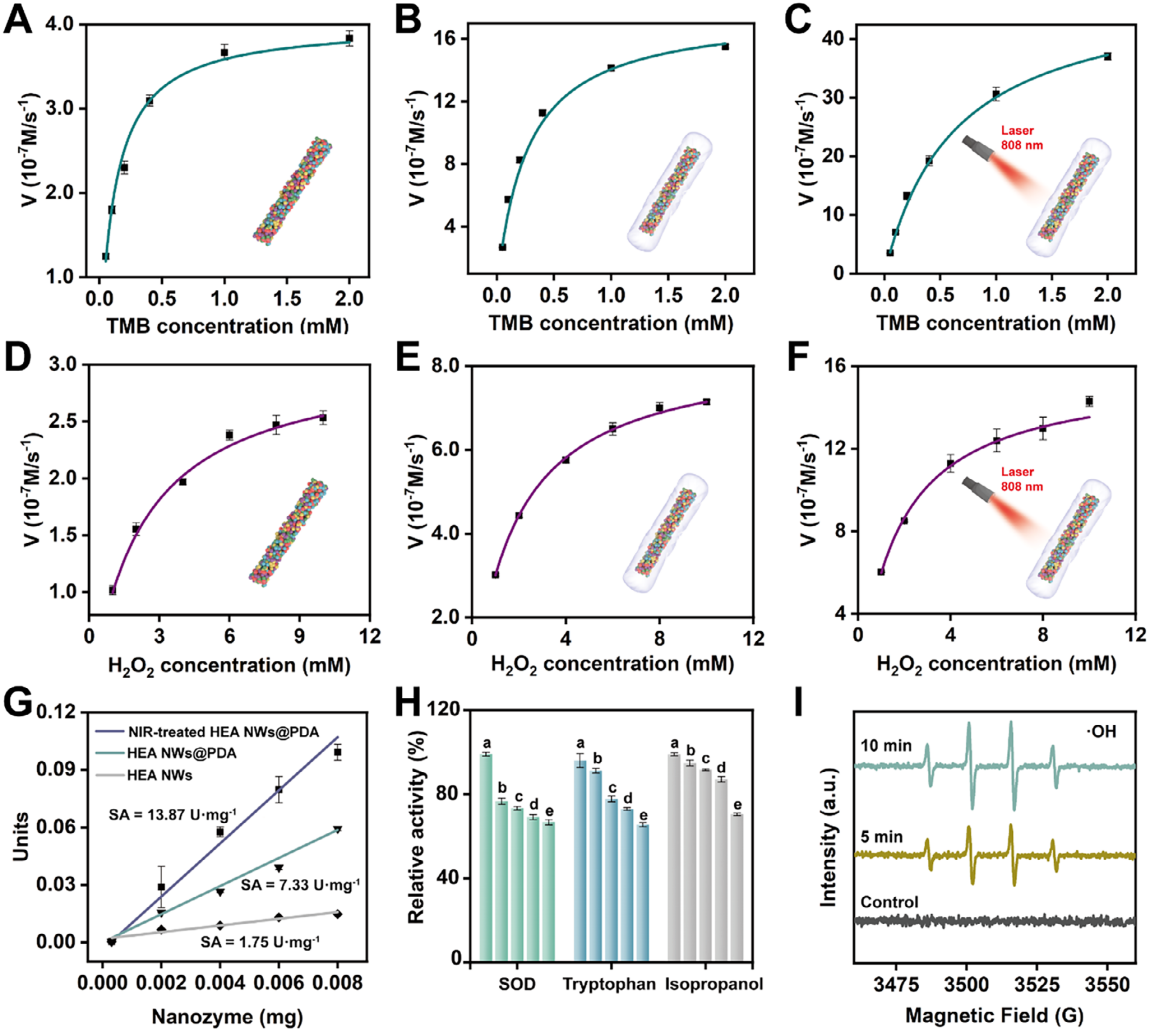
Michaelis‒Menten curves and POD mechanism of HEA NWs, HEA NWs@PDA, and NIR‐treated HEA NWs@PDA. A‒C) Michaelis‒Menten curves of HEA NWs, HEA NWs@PDA, and NIR‐treated HEA NWs@PDA with different concentrations of TMB. The data are presented as the mean ± SD (n = 6 independent samples). D‒F) Michaelis‒Menten curves of HEA NWs, HEA NWs@PDA, and NIR‐treated HEA NWs@PDA with different concentrations of H_2_O_2_. The data are presented as the mean ± SD (n = 6 independent samples). G) Specific activity of HEA NWs, HEA NWs@PDA, and NIR‐treated HEA NWs@PDA. The data are presented as the mean ± SD (n = 6 independent samples). H) Relative activity of the HEzymes@PDA+TMB system containing different scavengers. For SOD (a−e: 0, 5, 10, 15, and 20 U/L), tryptophan (a−e: 0, 0.5, 1.0, 1.5, and 2.0 mm), and isopropanol (a−e: 0, 0.1, 0.5, 1.0, and 1.5 mm). The data are presented as the mean ± SD (n = 6 independent samples). I) ESR spectra showing •OH formed from H_2_O_2_.

To evaluate the effect of PDA‐wrapped HEzymes and NIR irradiation on the POD‐like activity, enzymatic kinetics were assessed by varying the concentrations of TMB (**Figure** **4**A‒C) and H_2_O_2_ (Figure 4D‒F). Curves of the substrate concentration versus the initial reaction rate were obtained, which conformed to the classic Michaelis–Menten model, demonstrating that the POD‐like catalysis process complied with Michaelis kinetics.^[^ ^37^ ^]^

(1) $$v=\frac{V_{max}\left[S\right]}{K_{m}+\left[S\right]}$$

(2) $$\frac{1}{v}=\frac{K_{m}}{V_{max}\left[S\right]}+\frac{1}{V_{max}}$$

Using the equations above, a Lineweaver–Burk double reciprocal plot was constructed, enabling the determination of the Michaelis constant (*K_m_*) and maximum reaction rate (*V_max_*) for the respective substrates. In addition, the catalytic constant (*K_cat_*) and catalytic efficiency (*K_cat_/K_m_*) were derived (Figures S20 and S21, Supporting Information). It is widely acknowledged that the first critical step in the catalytic mechanism of nanomaterials mimicking POD‐like activity involves the reactive adsorption of H_2_O_2_ onto the nanozyme surface, followed by its catalytic decomposition to release free radicals, which subsequently drive the catalytic reaction of the chromogenic substrate.^[^ ^38^ ^]^ Thus, designing nanozymes with enhanced affinities for H_2_O_2_ is crucial.

Compared with HEA NWs, HEA NWs@PDA had a lower *K_m_* and higher *V_max_* for H_2_O_2_, which robustly indicated that the PDA coating enhanced the affinity of HEzymes for H_2_O_2_ and improved the catalytic rate (Table S1, Supporting Information).^[^ ^39^ ^]^ More crucially, HEA NWs@PDA subjected to NIR irradiation displayed a superior affinity and catalytic efficiency compared with those of HEA NWs@PDA alone. In addition, *K_cat_* reflected the ability of a single enzyme molecule to catalyze the transformation of substrates into products per unit of time, with higher *K_cat_* values indicating greater intrinsic catalytic activities. On the other hand, *K_cat_/K_m_* quantified the efficiency of substrate conversion per second by each enzyme molecule under substrate saturation conditions.^[^ ^40^ ^]^ Compared with HEA NWs, the *K_cat_* and *K_cat_/K_m_* of HEA NWs@PDA for H_2_O_2_ increased by 5.88 and 6.87 times, respectively. In particular, the *K_cat_* and *K_cat_/K_m_* of HEA NWs@PDA were further enhanced by 1.87 and 2.06 times under NIR irradiation (Table S1, Supporting Information). These results indicated that the catalytic ability and POD‐like activity of HEzymes could be significantly enhanced by PDA modification and NIR irradiation. In addition, a comparison of the kinetic parameters for TMB (Table S2, Supporting Information) revealed that the *V_max_*, *K_cat_*, and *K_cat_/K_m_* of NIR‐treated HEA NWs@PDA were the highest, followed by those of HEA NWs@PDA, with the HEA NWs exhibiting the lowest values. These results indicated that both PDA modification and NIR irradiation promoted the catalytic reaction of TMB. Furthermore, HEA NWs@PDA exhibited superior catalytic efficiency compared to previously reported nanozymes (Table S3, Supporting Information). The specific activities of the HEzymes were also evaluated, with values of 13.87 U mg^−1^ for NIR‐treated HEA NWs@PDA, 7.33 U mg^−1^ for HEA NWs@PDA, and 1.75 U mg^−1^ for HEA NWs. The enhanced performance of HEzymes@PDA under NIR irradiation highlighted the effectiveness of this approach (Figure 4G). Collectively, these results validated the feasibility of the stepwise enhancement of enzyme‐like activity through PDA modification and NIR‐assisted catalysis.

To better elucidate the catalytic mechanism of HEzymes@PDA, the reactive oxygen species (ROS) generated during the decomposition of H_2_O_2_ were investigated.^[^ ^41^ ^]^ Isopropanol, tryptophan, and superoxide dismutase (SOD) were used as scavengers for •OH, ^1^O_2_, and •O_2_^−^, respectively (Figure 4H). The relative catalytic activity of HEzymes@PDA gradually decreased with increasing concentrations of these scavengers, indicating the presence of •OH, ^1^O_2_, and •O_2_^−^ in the catalytic process. For a detailed investigation of the free radicals, electron spin resonance (ESR) spectroscopy was employed to identify the specific free radicals produced during the catalytic reaction. The results proved that •OH, ^1^O_2_, and •O_2_^−^ were generated, with signal intensities gradually strengthening over time, which mutually verified the results of the free‐radical scavenger experiments (Figure 4I; Figure S22, Supporting Information). In addition, in the absence of H_2_O_2_, the signals of the three free radicals were nearly undetectable, indicating that HEzymes@PDA exhibited a negligible OXD‐like activity. This observation aligned with the earlier results from pH optimization experiments.

### 2.5 DFT Calculations of HEzymes and HEzymes@PDA

**Figure 5:**
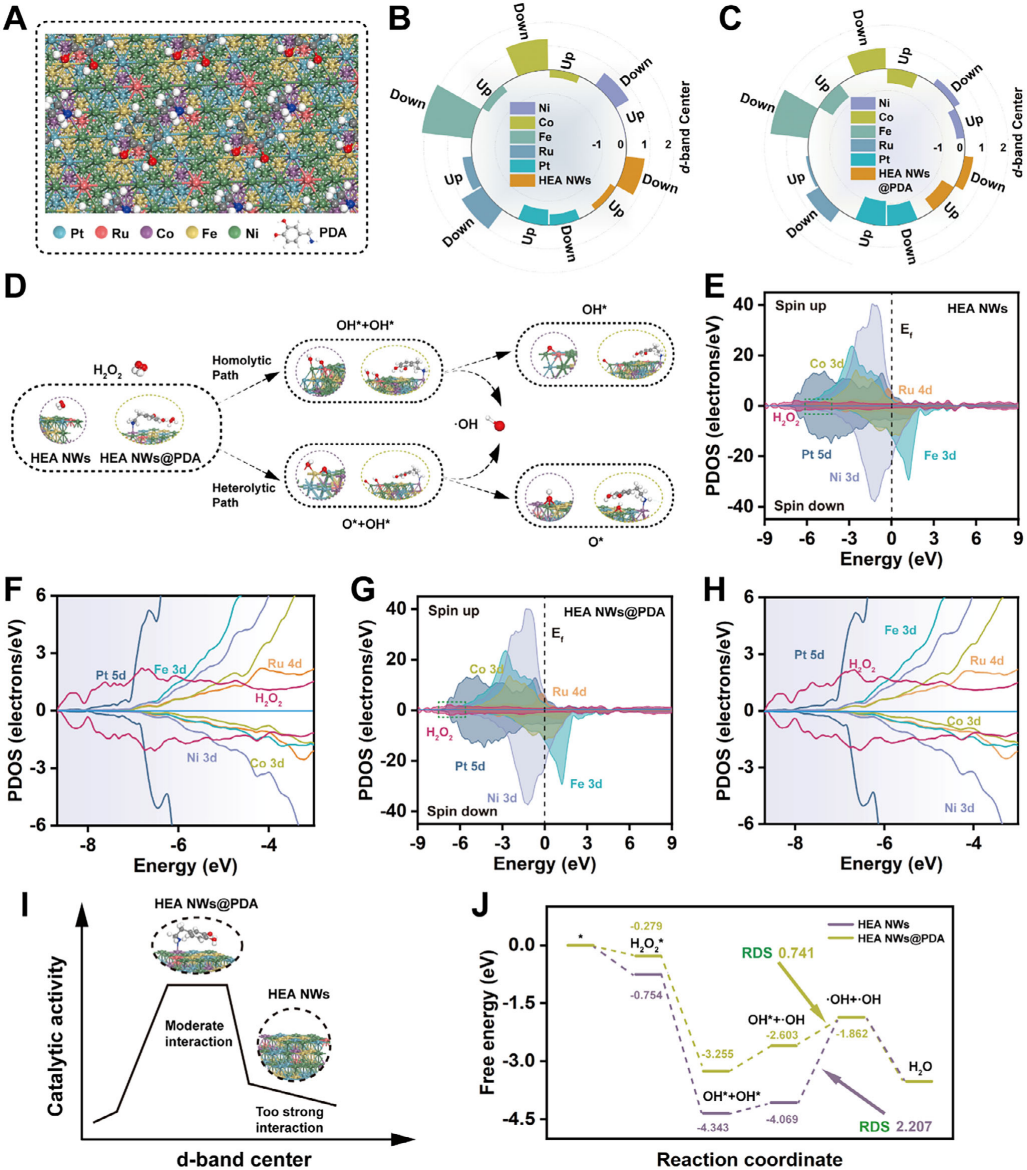
DFT calculations of the electron distribution and structural configuration, and theoretical calculations of the POD‐like mechanism of HEA NWs and HEA NWs@PDA. A) 3D atomic model showing the crystal structure of HEA NWs@PDA. B) The d‐band center comparisons for the individual elements and the bulk HEA NWs. C) The d‐band center comparisons for the individual elements and the bulk HEA NWs@PDA. D) Different reaction pathways for the decomposition of H_2_O_2_ to generate •OH on the HEA NWs and HEA NWs@PDA. E,F) PDOS and partial enlargement for H_2_O_2_ adsorption on the HEA NWs. G,H) PDOS and partial enlargement for H_2_O_2_ adsorption on the HEA NWs@PDA. I) Schematic illustration of Sabatier principle for HEA NWs and HEA NWs@PDA. J) Optimized free energy profiles for H_2_O_2_ decomposition along homolytic pathways on HEA NWs and HEA NWs@PDA.

The previous experiments demonstrated that PDA modification significantly enhanced the catalytic performance of HEA NWs. Notably, the catalytic activity of nanozymes is closely associated with their surface properties and the electronic structures of their active sites. To elucidate the mechanism underlying the enhanced POD‐like activity of HEA NWs@PDA, DFT calculations were conducted on both HEA NWs and HEA NWs@PDA.^[^ ^42^ ^]^ Leveraging on the relevant characterization results, such as ICP‐OES and XRD, the most stable and energetically favorable structure of HEA NWs, along with the corresponding reaction sites, was selected as the theoretical model for the study (Figures S23‒S26A, Supporting Information). Using this as a foundation, a model for HEA NWs@PDA was designed (**Figure** **5**A; Figure S26B, Supporting Information). The total density of states (TDOS) analysis revealed that both HEA NWs and HEA NWs@PDA exhibited significant electron distributions near the Fermi level (E_f_) (Figure S27, Supporting Information). The electronic states around the E_f_ influenced the adsorption and catalytic capabilities of the HEA NWs toward the reactants. After modification with PDA, the electronic structure of the HEA NWs underwent a discernible change, featuring a higher electron abundance at the E_f_. This indicated that there were more active electrons on the surface of HEA NWs@PDA, which facilitated a higher catalytic reaction rate.

The partially projected density of states (PDOS) of the HEA NWs and HEA NWs@PDA were utilized to analyze the catalytically active sites within their overall structures and disclose the synergistic interactions among the different metals (Figure S28A,B, Supporting Information). A distinct overlap was observed among the d‐orbitals of the various metals, indicating strong bonding interactions. The three transition metals (Fe, Co, and Ni) displayed a broad frequency band in the middle region of the spectrum, with notable orbital overlaps, verifying the strong coupling among the 3‐d orbitals of these transition metals. Moreover, the Pt‐5d orbital was located at ≈−5 eV, which was the farthest from the E_f_, indicating that Pt on the surface acted as an electron reservoir.^[^ ^15^ ^]^ This role helped to maintain the balance of valence states within the HEA NWs during the catalytic process. However, the highest peak of Ru appeared at −2.2 eV, which was the closest to the E_f_. Pt also exhibited a sharp peak near the E_f_. The electronic structure of the Pt–Ru bimetallic sites significantly improved the electron density at the E_f_, enhancing the adsorption and transfer efficiency of the substrate.^[^ ^43^ ^]^ Thus, the synergistic complementarity between the Fe and Co–Ni transition metals and the Pt–Ru noble metals induced lattice distortion and created uneven bonding orbitals in the HEA NWs. Through electron transfer and orbital hybridization, the HEA NWs provided numerous active sites for catalytic activity.^[^ ^44^ ^]^ Moreover, HEA NWs@PDA exhibited a similar trend in the distribution of metal elements, confirming that the internal element bonding and synergistic interaction of the HEA NWs were not influenced by the PDA modification (Figure S28B, Supporting Information).

The d‐band centers of the overall structures of the HEA NWs, HEA NWs@PDA, and five internal metal elements are clearly depicted in Figure 5B,C. According to the d‐band center theory, for HEA NWs, Ru (spin up: 0.39 eV, spin down: 0.98 eV) with a higher d‐band center position has stronger adsorption of reactants. This is because the electron cloud was farther from the electron nucleus, making it more “diffused” and, therefore, more likely to react with molecules. Crucially, the d‐band center of Pt was located at a lower energy level (spin up: −0.656 eV, spin down: −0.446 eV), indicating a higher electron cloud density. This signified that Pt was more likely to provide electrons during the catalytic reaction, facilitating electron transfer. This behavior aligned with its role as an electron reservoir. Adhering to the Hammer–Nørskov d‐band model, the d‐band center of a metal determines the position of electron donor–acceptor interactions. The d‐band centers of the Pt–Ru dual‐site differed significantly, with the Ru center having the highest energy and Pt center having the lowest. This energy difference provided the driving force for electron transfer from Pt to Ru, and the self‐complementary induction between these two metals allowed the substrate and its intermediates to adsorb more stably.^[^ ^13b^ ^]^ The three transition metals (Fe, Co, and Ni) had higher energies in the spin‐down state and lower energies in the spin‐up state. These transition metals worked synergistically with Pt–Ru to promote electron transfer, thereby enhancing catalytic effects. After PDA modification, although the trend of the d‐band centers of the metals in the overall catalyst remained unchanged, the d‐band centers of both the overall structure and five metals of HEA NWs@PDA became more negative than those of the unmodified HEA NWs. This downward shift of the d‐band center reduced the number of antibonding states generated by coupling, causing more antibonding states to fall below the Fermi level and become electron‐filled. This destabilized the bonding states, reducing the adsorption energy and making it easier to dissociate and transform the substrate through stable adsorption compared with HEA NWs. In addition, the more negative d‐band center of HEA NWs@PDA altered the donor–acceptor properties of the substrate, enabling it to provide/accept more electrons and enhance electron transfer, which facilitated catalytic reactions. In summary, PDA modification did not influence the internal structure of the HEA NWs. Conversely, it fine‐tuned the d‐band center of the HEA NWs and optimized the surface electron cloud density, substantially improving their catalytic capability.^[^ ^45^ ^]^

The mechanisms underlying the POD‐like activities of the HEA NWs and HEA NWs@PDA were further explored. According to previous studies, there are two splitting mechanisms for the catalytic decomposition of H_2_O_2_: homolytic and heterolytic.^[^ ^46^ ^]^ Figure 5D and Figures S29‒S33 (Supporting Information) show the homolytic and heterolytic decomposition of H_2_O_2_ catalyzed by the HEA NWs and HEA NWs@PDA, which ultimately generate •OH to catalyze the oxidation of TMB. We first analyzed the TDOS profiles of the HEA NWs and HEA NWs@PDA after the adsorption of H_2_O_2_ (Figures S34 and S35, Supporting Information). The electron density of HEA NWs@PDA at the E_f_ was higher than that of the HEA NWs. Similar results were observed in the TDOS data of HEA NWs@PDA and the HEA NWs after the decomposition and adsorption of •OH. These findings proved that HEA NWs@PDA had more electronic states available for electron occupation and transition than the HEA NWs. To assess the adsorption capacity of the HEA NWs and HEA NWs@PDA for H_2_O_2_ in more detail, the PDOS diagrams clearly indicated that, when H_2_O_2_ was adsorbed on the surfaces of the HEA NWs and HEA NWs@PDA, the s‐ and p‐orbitals of the adsorbed H_2_O_2_ exhibited significant overlap with the d‐orbitals of the adjacent metals, suggesting that both catalysts could stably adsorb H_2_O_2_ (Figure 5E‒H). In addition, the adsorption of the intermediate •OH on the HEA NWs and HEA NWs@PDA displayed similar results (Figure S36, Supporting Information).

Based on the dissociation mechanism of H_2_O_2_, we calculated the Gibbs free energy changes at each step of the catalytic process, providing a more intuitive comparison of the activities of the two catalysts. As shown in Figure 5J, both the HEA NWs and HEA NWs@PDA exhibited negative adsorption energies (HEA NWs: −0.754 eV, HEA NWs@PDA: −0.279 eV), further proving that both catalysts could stably adsorb H_2_O_2_. According to Sabatier principle, the adsorption of reactants onto the surface of an ideal catalyst should not be too strong or too weak. Excessive adsorption could cause the reactants to adsorb too tightly, hindering product desorption, while weak adsorption would undermine the catalyst's ability to adsorb reactants. The PDA modification shifted the d‐band center to a more negative value, thereby optimizing the adsorption of reactants and intermediates. This adjustment ensured that the adsorption was neither too strong to prevent decomposition and dissociation, nor too weak to impede effective catalysis, thereby achieving the optimal catalytic state (Figure 5I).^[^ ^24^ ^]^ Subsequently, the peroxide bond in H_2_O_2_ was uniformly broken, generating two •OH radicals (Figure 5J). The rate‐determining step (RDS) for the catalytic decomposition of H_2_O_2_, which represented the slowest step of the reaction, was crucial for guaranteeing the catalytic reaction rate. The superiority of the catalysts was reflected in the reduction in their RDS activation energies, specifically the step with the highest energy barrier for generating •OH from the OH* intermediate. The energy barrier for the RDS in the homogeneous cleavage of H_2_O_2_ catalyzed by HEA NWs@PDA to generate •OH was significantly lower than that of the HEA NWs (HEA NWs@PDA, ∆E = 0.741; HEA NWs, ∆E = 2.207), which accounted for the faster catalytic rate of HEA NWs@PDA. We also investigated the free‐energy alterations at each step of the heterogeneous decomposition of H_2_O_2_ catalyzed by the two catalysts (Figure S37, Supporting Information). Similarly, the energy barrier for generating the first •OH by HEA NWs@PDA was higher than those of other reaction steps (∆E = 1.062), serving as the RDS for catalyzing the heterogeneous decomposition of H_2_O_2_. For HEA NWs, the energy required for the RDS in the heterogeneous decomposition of H_2_O_2_ was higher compared with that of HEA NWs@PDA (∆E = 1.785). This further demonstrated that HEA NWs@PDA possessed a superior catalytic performance compared with that of the HEA NWs in catalyzing the heterogeneous decomposition of H_2_O_2_.^[^ ^47^ ^]^

Overall, the RDS of the HEA NWs undergoing heterolytic cleavage was relatively lower than that of the HEA NWs undergoing homolytic cleavage, indicating that the HEA NWs may exert a catalytic effect by catalyzing the heterolytic cleavage of H_2_O_2_ to generate •OH. However, for HEA NWs@PDA, the RDS of homolytic cleavage was lower than that of heterolytic cleavage, suggesting that the catalysis of H_2_O_2_ homolytic cleavage played a dominant role. Regardless of whether homolytic or heterolytic cleavage occurred, the energy barrier required for the RDS of H_2_O_2_ decomposition catalyzed by HEA NWs@PDA was smaller than that of HEA NWs. The DFT results comprehensively clarified the relationship between the improved catalytic performance by PDA modification and Sabatier principle. Specifically, by optimizing the charge distribution on the surface of the HEA NWs and appropriately reducing the energy of the d‐band center to balance product coverage, PDA modification significantly elevated the adsorption–dissociation efficiency and POD‐like activity of HEA NWs@PDA.

### 2.6 Application of HEzymes@PDA in Biosensing

**Figure 6:**
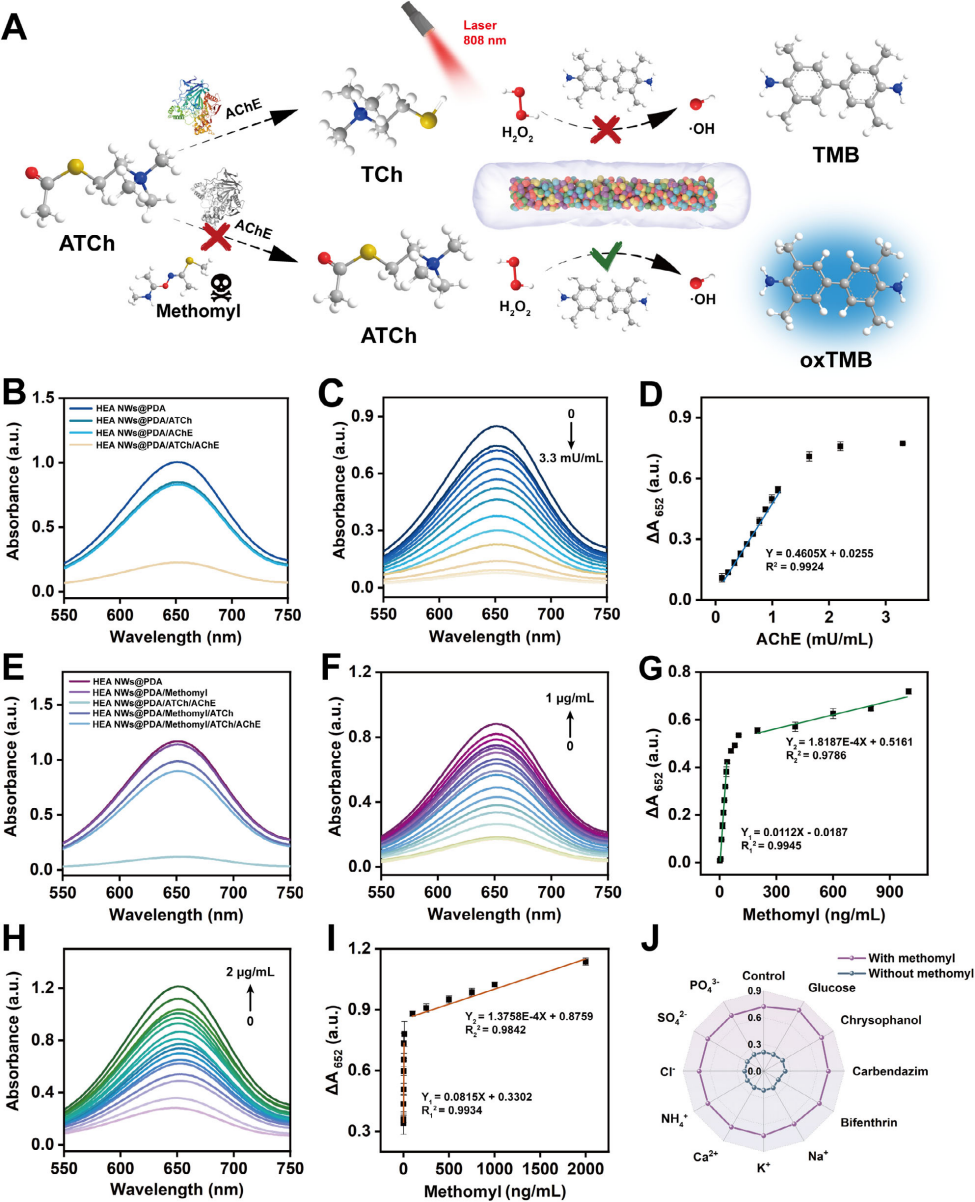
The colorimetric detection platform for AChE and methomyl. A) Illustration of the detection mechanism for AChE and methomyl based on AChE‐controlled chromogenic reaction of the HEzymes@PDA/TMB/H_2_O_2_ system. B) UV–vis absorption spectra of HEzymes@PDA/TMB/H_2_O_2_ systems with different compounds to investigate the feasibility of detecting AChE. C) UV–vis absorption spectra of colorimetric solution influenced by AChE concentrations. D) The relationship of absorbance change at 652 nm with AChE concentration and corresponding linear relationship. E) UV–vis absorption spectra of HEzymes@PDA/TMB/H_2_O_2_ systems with different compounds to investigate the feasibility of detecting methomyl. F) UV–vis absorption spectra of colorimetric solution influenced by methomyl concentrations. G) The relationship of absorbance change at 652 nm with methomyl concentration and corresponding linear relationship. H) UV–vis absorption spectra of colorimetric solution influenced by methomyl concentrations under NIR light. I) The relationship of absorbance change at 652 nm with methomyl concentration and corresponding linear relationship under NIR light. J) Interference experiment of methomyl colorimetric detection platform.

Acetylcholinesterase (AChE) plays a crucial role in terminating the signal transmission of neurotransmitters in the synaptic cleft and is widely used as a biomarker for disease diagnosis.^[^ ^48^ ^]^ Acetylthiocholine (ATCh), an AChE substrate, decomposes to generate thiocholine (TCh). Since TCh is a potent reducing agent, it can reduce blue oxTMB to colorless TMB, leading to a decrease in color intensity and absorbance.^[^ ^49^ ^]^ Building on this principle, we explored the potential applications of HEzymes@PDA by constructing a biosensor for the visual detection of AChE (**Figure** **6**A). As shown in Figure 6B, in the presence of both ATCh and AChE, the absorbance of the chromogenic system at 652 nm decreased significantly, whereas ATCh or AChE alone had little influence on the catalytic activity of HEzymes@PDA. As the AChE concentration increased, the absorbance of oxTMB gradually decreased (Figure 6C). In addition, within the range of 0.1‒1.1 mU mL^−1^ AChE, a favorable linear relationship was observed with an absorbance change (∆A_652_) (∆A_652_ = 0.4605 × C_AChE_ + 0.0255, R^2^ = 0.9924), and the limit of detection (LOD) was 0.064 mU mL^−1^ (Figure 6D). Compared with previously reported sensors for AChE detection, the HEzymes@PDA biosensor displayed an ultra‐low LOD and high sensitivity (Table S4, Supporting Information). In addition, the specificity of the proposed biosensor for detecting AChE was investigated. Common proteins, including horseradish peroxidase, glucose oxidase, neprilysin, acid phosphatase, papain, and bovine serum albumin, were selected as potential interfering substances. In the presence or absence of AChE, the influence of these proteins on the absorbance of the colorimetric system was negligible (Figure S38, Supporting Information), suggesting that this sensing platform had excellent specificity for AChE detection.

Carbamate pesticides are widely used to prevent and control plant diseases, pests, and weeds.^[^ ^50^ ^]^ However, these compounds accumulate in the environment and damage the human body by inhibiting AChE.^[^ ^51^ ^]^ Thus, rapid and sensitive detection of carbamate pesticide residues is important for ensuring human health and food safety.^[^ ^52^ ^]^ Based on the inhibitory action of carbamate pesticides on AChE, we chose methomyl as a typical model and combined it with the HEzymes@PDA/AChE biosensor. As shown in Figure 6E, adding only methomyl had minimal effect on the absorbance, excluding its interference with the reaction system. As the concentration of methomyl increased, the activity of AChE was inhibited, and the decreased absorbance was subsequently restored (Figure 6F). This biosensing platform showed two favorable linear relationships within the concentration ranges of 1‒40 and 200‒1000 ng mL^−1^ for methomyl, with linear equations of ∆A_652_ = 0.0112 × C_methomyl_ – 0.0187 (R^2^ = 0.9945) and ∆A_652_ = 1.8187×10^−4^ × C_methomyl_ + 0.5161, (R^2^ = 0.9786), as well as an LOD of 0.329 ng mL^−1^ (Figure 6G). This probe exhibited a superior sensing performance compared with those of other methods for detecting methomyl (Table S5, Supporting Information). Considering the complexity of the actual sample, other pesticides and various ions were selected as interfering substances to evaluate the specificity of the sensor for methomyl assays (Figure 6J). Similarly, this sensor demonstrated considerably high selectivity in the presence or absence of these interfering substances.

In addition, to explore the latent role of the photothermal effect in methomyl monitoring, the same process was conducted under NIR‐assisted conditions. The sensing platform based on NIR‐treated HEzymes@PDA showed strong linear relationships in the range of 0.1‒5 and 100‒2000 ng mL^−1^ after NIR irradiation, with linear equations of ∆A_652_ = 0.0815 × C_methomyl_ + 0.3302 (R^2^ = 0.9934) and ∆A_652_ = 1.3758×10^−4^ × C_methomyl_ + 0.8759 (R^2^ = 0.9842). The LOD was only 0.0628 ng mL^−1^, which was 5 times lower than that without NIR irradiation (Figure 6H,I). These results demonstrated that the photothermal effect could significantly improve the sensitivity of methomyl detection by enhancing the catalytic performance of HEzymes@PDA.^[^ ^53^ ^]^

### 2.7 A Portable Sensing Platform Based on Smartphone‐Assisted Hydrogel@HEzymes@PDA

**Figure 7:**
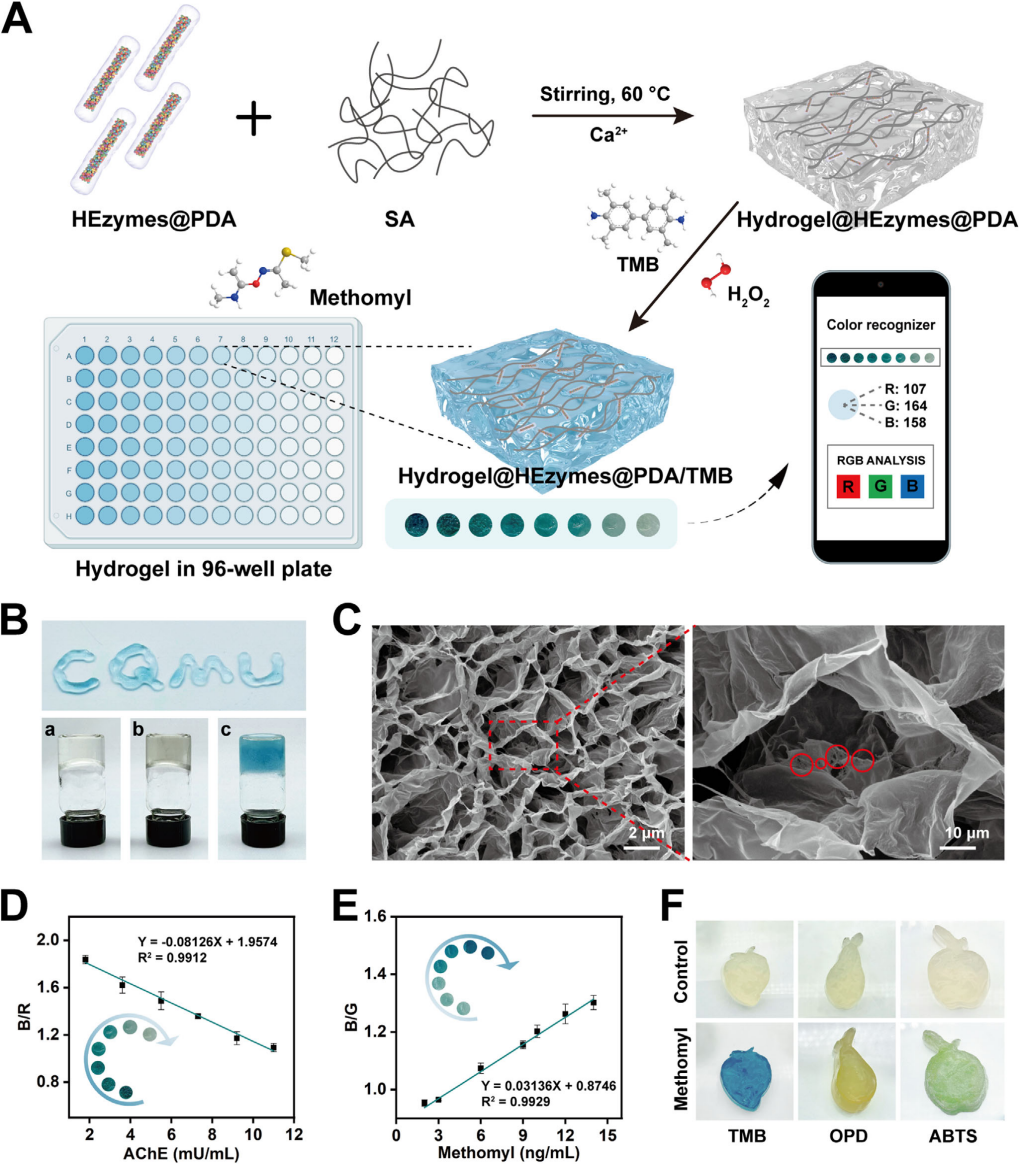
Synthesis of Hydrogel@HEzymes@PDA and construction of portable sensing platform. A) Schematic diagram of the synthesis of Hydrogel@HEzymes@PDA and constructing a visual methomyl detection platform. B) Digital photos of hydrogels (a, SA hydrogel; b, Hydrogel@HEzymes@PDA; c, Hydrogel@HEzymes@PDA/TMB) under white light. C) SEM images of Hydrogel@HEzymes@PDA. The red circles indicate that HEzymes@PDA are located inside the hydrogel. D) The relationship of B/R with AChE concentration and the corresponding linear relationship. The inner shows the digital photos of different concentrations of AChE detected by Hydrogel@HEzymes@PDA. E) The relationship of B/G with methomyl concentration and the corresponding linear relationship. The inner shows the digital photos of different concentrations of methomyl detected by Hydrogel@HEzymes@PDA. F) Digital photos of Hydrogel@HEzymes@PDA with TMB, OPD, and ABTS in different interesting shapes (strawberry, pear, and apple) under white light.

Sodium alginate (SA) is a natural, seaweed‐derived polysaccharide that contains numerous carboxyl groups, offering low irritation and toxicity. SA can cross‐link with Ca^2+^ to form a 3D hydrogel structure, which can effectively immobilize nanozymes and chromogenic reagents while aiding in size sieving and color‐producing stability.^[^ ^54^ ^]^ Moreover, the high hydrophilicity and hydration capacity of SA enhance the sensitivity and speed of nanozyme responses to target analytes. Leveraging these advantages, we constructed a portable, high‐throughput visual sensing platform using SA hydrogels to encapsulate HEzymes@PDA (Hydrogel@HEzymes@PDA) within 96‐well plates. This platform was combined with smartphone‐based color recognition to ensure more accurate visual detection (**Figure** **7**A). Figure 7B shows the construction and injectability of the SA hydrogel and Hydrogel@HEzymes@PDA. A discernible color change was observed upon adding TMB and H_2_O_2_, confirming the POD‐like activity and colorimetric capability of Hydrogel@HEzymes@PDA and validating its feasibility for colorimetric detection. The internal morphology of the SA hydrogel was revealed through scanning electron microscopy (SEM), showing a well‐defined porous structure (Figure S39, Supporting Information). Similarly, the SEM images of Hydrogel@HEzymes@PDA displayed a comparable porous reticular structure (Figure 7C). Upon magnification, the HEA NWs@PDA loading was clearly visible within the cavities. Benefiting from the ultrasmall nanostructure and PDA modification, HEzymes@PDA could be loaded relatively quickly without disrupting the original organizational structure of the SA hydrogel. This structural compatibility served as a foundation for improving the catalytic performance of Hydrogel@HEzymes@PDA.

The SA hydrogel loaded with HEzymes@PDA was incorporated into 96‐well plates, where specific reactions were initiated by adding substrates, resulting in progressively attenuating colorimetric signals. These signals were captured using a smartphone camera, and the color information of the images was processed through RGB analysis. As depicted in Figure 7D, the B/R value exhibited an excellent linear relationship with AChE concentrations ranging from 1.8 to 11 mU mL^−1^ (B/R = −0.08126 × C_AChE_ + 1.9574, R^2^ = 0.9912). Similarly, as the methomyl concentration increased, the color development of Hydrogel@HEzymes@PDA gradually recovered (Figure 7E). The B/G value demonstrated a favorable linear range with methomyl concentrations in the range of 2.0‒14 ng mL^−1^ (B/G = 0.03136 × C_methomyl_ + 0.8746, R^2^ = 0.9929). These results confirmed that the visualized biosensing platform based on Hydrogel@HEzymes@PDA possessed considerable feasibility for detecting AChE and methomyl. Interestingly, the 3D network structure of the SA hydrogel, combined with the flexibility of its molecular chains, allowed Hydrogel@HEzymes@PDA to be cross‐linked and solidified into various molds, producing hydrogel products shaped according to the mold design.^[^ ^55^ ^]^ Given methomyl's predominant use in agriculture, Hydrogel@HEzymes@PDA was fabricated into shapes resembling strawberries, pears, and apples, employing TMB, OPD, and ABTS as chromogenic substrates. These hydrogels were successfully applied for methomyl detection (Figure 7F).^[^ ^56^ ^]^ Consequently, the constructed Hydrogel@HEzymes@PDA sensor fulfilled the requirements for the on‐site visual detection of AChE and methomyl in diverse scenarios.

To evaluate the practical applicability of Hydrogel@HEzymes@PDA, three types of fruits were selected as real samples to assess the performance of this sensor in detecting residual methomyl. Different concentrations of methomyl were added to the pretreated samples, and the recovery rates were determined (Table S6, Supporting Information). For strawberries, pears, and apples, the recovery rates ranged from 95.6% to 102.0%, with a relative standard deviation (RSD) value of <3.16%. These results proved that the Hydrogel@HEzymes@PDA sensing platform was suitable for the on‐site detection of methomyl in actual samples and demonstrated great potential for biological applications.

## 3 Conclusion

We developed a novel HEzymes@PDA catalyst with efficient POD‐like catalytic activity and remarkable photothermal properties by employing surface‐engineering techniques to modulate PtRuFeCoNi HEA NWs. The self‐polymerized PDA coating significantly enhanced the catalytic performance of the HEAs. Under NIR irradiation, HEzymes@PDA accelerated the formation and diffusion of free radicals, greatly improving the catalytic rate. DFT analysis showed that the Pt–Ru bimetal had strong substrate adsorption owing to site‐to‐site electron transfer between the bimetal and transition metal. The Sabatier principle comprehensively explained the high catalytic efficiency of HEzymes@PDA: PDA‐induced downward shifting of the d‐band center of the HEAs decreased the adsorption energy while increasing the surface electron density, thereby enhancing the electron mobility and optimizing substrate adsorption and product desorption. A HEzymes@PDA biosensor was successfully constructed, enabling the rapid visual detection of AChE and methomyl in fruits when combined with hydrogel technology. In summary, this study integrates surface‐engineering strategies into the HEzyme field, using the Sabatier principle to establish the relationship between the d‐band center location and substrate adsorption and desorption abilities of the catalysts. This approach offers a new perspective for the design and biological applications of HEzyme complexes.

## Conflict of Interest

The authors declare no conflict of interest.

## Supporting information



Supporting Information

## Data Availability

The data that support the findings of this study are available from the corresponding author upon reasonable request.

## References

[1] a) J. Wu , X. Wang , Q. Wang , Z. Lou , S. Li , Y. Zhu , L. Qin , H. Wei , Chem. Soc. Rev. 2019, 48, 1004; 30534770 10.1039/c8cs00457a

[2] a) Y. Huang , J. Ren , X. Qu , Chem. Rev. 2019, 119, 4357; 30801188 10.1021/acs.chemrev.8b00672

[3] a) J. Sheng , Y. Wu , H. Ding , K. Feng , Y. Shen , Y. Zhang , N. Gu , Adv. Mater. 2024, 36, 2211210; 10.1002/adma.202211210 36840985

[4] a) Y. Zhang , G. Wei , W. Liu , T. Li , Y. Wang , M. Zhou , Y. Liu , X. Wang , H. Wei , Nat. Rev. Methods Primers 2024, 4, 36;

[5] a) Y. Sun , B. Xu , X. Pan , H. Wang , Q. Wu , S. Li , B. Jiang , H. Liu , Coord. Chem. Rev. 2023, 475, 214896;

[6] Z. Wang , R. Zhang , X. Yan , K. Fan , Mater. Today 2020, 41, 81.

[7] a) Z. Li , K. G. Pradeep , Y. Deng , D. Raabe , C. C. Tasan , Nature 2016, 534, 227; 27279217 10.1038/nature17981

[8] a) C.‐Y. Cheng , Y.‐C. Yang , Y.‐Z. Zhong , Y.‐Y. Chen , T. Hsu , J.‐W. Yeh , Curr. Opin. Solid State Mater. Sci. 2017, 21, 299;

[9] a) H. Cheng , H. Luo , Z. Pan , X. Wang , Q. Zhao , Y. Fu , X. Li , J. Mater. Sci. Technol. 2023, 155, 211;

[10] W.‐L. Hsu , C.‐W. Tsai , A.‐C. Yeh , J.‐W. Yeh , Nat. Rev. Chem. 2024, 8, 471. 38698142 10.1038/s41570-024-00602-5

[11] a) Y. Xin , S. Li , Y. Qian , W. Zhu , H. Yuan , P. Jiang , R. Guo , L. Wang , ACS Catal. 2020, 10, 11280;

[12] Y. Sun , W. Zhang , Q. Zhang , Y. Li , L. Gu , S. Guo , Matter 2023, 6, 193.

[13] a) X. Huang , G. Yang , S. Li , H. Wang , Y. Cao , F. Peng , H. Yu , J. Energy Chem. 2022, 68, 721;

[14] T. Yang , J. Huang , S. Hu , Z. Zhang , Z. Ma , S. Liu , Y. Liu , B. Ge , P. K. Shen , S. Gao , Chem. Eng. J. 2024, 500, 157426.

[15] C. Zhan , Y. Xu , L. Bu , H. Zhu , Y. Feng , T. Yang , Y. Zhang , Z. Yang , B. Huang , Q. Shao , Nat. Commun. 2021, 12, 6261. 34716289 10.1038/s41467-021-26425-2 PMC8556242

[16] a) T. A. Batchelor , J. K. Pedersen , S. H. Winther , I. E. Castelli , K. W. Jacobsen , J. Rossmeisl , Joule 2019, 3, 834;

[17] a) Y. Ai , M. Q. He , H. Sun , X. Jia , L. Wu , X. Zhang , H. b. Sun , Q. Liang , Adv. Mater. 2023, 35, 2302335; 10.1002/adma.202302335 36995655

[18] a) S. Ji , B. Jiang , H. Hao , Y. Chen , J. Dong , Y. Mao , Z. Zhang , R. Gao , W. Chen , R. Zhang , Nat. Catal. 2021, 4, 407;

[19] a) X. Li , W. Tan , J. Fan , K. Li , ACS Nano 2024, 18, 24162; 39162692 10.1021/acsnano.4c05516

[20] S. Gao , D. Zhang , M. Pedrero , Z. Guo , J. M. Pingarrón , S. Campuzano , X. Zou , Coord. Chem. Rev. 2024, 501, 215564.

[21] H. A. Lee , Y. Ma , F. Zhou , S. Hong , H. Lee , Acc. Chem. Res. 2019, 52, 704. 30835432 10.1021/acs.accounts.8b00583

[22] G. Ma , X. Zhang , K. Zhao , S. Zhang , K. Ren , M. Mu , C. Wang , X. Wang , H. Liu , J. Dong , ACS Nano 2024, 18, 3369. 38251846 10.1021/acsnano.3c10249

[23] a) C. Zhang , W. Liu , Z. Li , B. Yan , J. Lin , C. Chen , L. Zhang , Y. Lu , ACS Sustainable Chem. Eng. 2022, 10, 1653;

[24] J. Tian , Y. Rao , W. Shi , J. Yang , W. Ning , H. Li , Y. Yao , H. Zhou , S. Guo , Angew. Chem., Int. Ed. 2023, 62, 202310894. 10.1002/anie.202310894 37698488

[25] Y. Shao , W. Xu , Y. Zheng , Z. Zhu , J. Xie , X. Wei , Y. Zhang , J. Zhang , Q. Wu , J. Wang , Chem. Eng. J. 2023, 455, 140586.

[26] L. Tao , M. Sun , Y. Zhou , M. Luo , F. Lv , M. Li , Q. Zhang , L. Gu , B. Huang , S. Guo , J. Am. Chem. Soc. 2022, 144, 10582. 35652187 10.1021/jacs.2c03544

[27] X. Ma , S. Zhang , Y. Zhou , W. Lei , Y. Zhai , Y. Zhao , C. Shan , J. Mater. Chem. A 2024, 12, 8862.

[28] Y. Chen , Y. Wang , Z. Chen , J. Cai , K. Li , H. Huang , F. Song , M. Gao , Y. Yang , L. Zheng , Mater. Today Nano 2022, 19, 100240.

[29] Y. Deng , W. Liu , R. Xu , R. Gao , N. Huang , Y. Zheng , Y. Huang , H. Li , X. Y. Kong , L. Ye , Angew. Chem., Int. Ed. 2024, 63, 202319216. 10.1002/anie.202319216 38337143

[30] Z. Hu , K. Chen , Y. Zhu , B. Liu , J. Shen , Small 2024, 20, 2309819. 10.1002/smll.202309819 38229574

[31] D. Wang , J. Wang , X. J. Gao , H. Ding , M. Yang , Z. He , J. Xie , Z. Zhang , H. Huang , G. Nie , Adv. Mater. 2024, 36, 2310033. 10.1002/adma.202310033 37994246

[32] a) P. Gong , C. Li , D. Wang , S. Song , W. Wu , B. Liu , J. Shen , J. Liu , Z. Liu , J. Colloid Interface Sci. 2023, 642, 612; 37028168 10.1016/j.jcis.2023.03.178

[33] G. Li , N. Marinkovic , B. Wang , M. R. Komarneni , D. E. Resasco , ACS Catal. 2022, 12, 13930.

[34] M. Yin , D. Lei , Y. Liu , T. Qin , H. Gao , W. Lv , Q. Liu , L. Qin , W. Jin , Y. Chen , J. Nanobiotechnol. 2024, 22, 321. 10.1186/s12951-024-02570-w PMC11162040 38849841

[35] Y. Zhu , Z. Wang , R. Zhao , Y. Zhou , L. Feng , S. Gai , P. Yang , ACS Nano 2022, 16, 3105. 35040328 10.1021/acsnano.1c10732

[36] J. Wu , Q. Liu , D. Jiao , B. Tian , Q. Wu , X. Chang , H. Chu , S. Jiang , Q. Yang , T. Liu , Angew. Chem., Int. Ed. 2024, 63, 202403203. 10.1002/anie.202403203 38590293

[37] J.‐J. Zheng , F. Zhu , N. Song , F. Deng , Q. Chen , C. Chen , J. He , X. Gao , M. Liang , Nat. Protoc. 2024, 19, 3470. 39147983 10.1038/s41596-024-01034-7

[38] L. Gao , J. Zhuang , L. Nie , J. Zhang , Y. Zhang , N. Gu , T. Wang , J. Feng , D. Yang , S. Perrett , Nat. Nanotechnol. 2007, 2, 577. 18654371 10.1038/nnano.2007.260

[39] M. Zhang , G. Li , X. Sun , Y. Jiang , X. Zhang , J. Mater. Chem. A 2017, 5, 20789.

[40] S. Wei , W. Ma , M. Sun , P. Xiang , Z. Tian , L. Mao , L. Gao , Y. Li , Nat. Commun. 2024, 15, 6888. 39134525 10.1038/s41467-024-51022-4 PMC11319669

[41] X. Wang , Q. Shi , Z. Zha , D. Zhu , L. Zheng , L. Shi , X. Wei , L. Lian , K. Wu , L. Cheng , Bioact. Mater. 2021, 6, 4389. 33997515 10.1016/j.bioactmat.2021.04.024 PMC8111038

[42] a) B. Wang , Y. Yao , X. Yu , C. Wang , C. Wu , Z. Zou , J. Mater. Chem. A 2021, 9, 19410;

[43] Y. Wei , X. Wu , Y. Zhao , L. Wang , Z. Zhao , X. Huang , J. Liu , J. Li , Appl. Catal., B 2018, 236, 445.

[44] Y. Wan , W. Wei , S. Ding , L. Wu , H. Qin , X. Yuan , Adv. Funct. Mater. 2024, 34, 2414554.

[45] L. Hao , X. J. Liang , Y. Zhang , Z. Zhang , Y. Han , Y. Jin , L. Li , A. Magrini , M. Bottini , S. Gao , Adv. Mater. 2024, 36, 2412368. 10.1002/adma.202412368 39396367

[46] Z. Yu , Z. Xu , R. Zeng , M. Xu , M. Zou , D. Huang , Z. Weng , D. Tang , Angew. Chem., Int. Ed. 2024, 64, 202420200. 10.1002/anie.202420200 39557613

[47] K. S. Exner , I. Sohrabnejad‐Eskan , H. Over , ACS Catal. 2018, 8, 1864.

[48] D.‐M. Liu , B. Xu , C. Dong , TrAC, Trends Anal. Chem. 2021, 142, 116320.

[49] W. Xiao , S. Cai , T. Wu , Z. Fu , X. Liu , C. Wang , W. Zhang , R. Yang , J. Colloid Interface Sci. 2023, 635, 481. 36599245 10.1016/j.jcis.2022.12.151

[50] F. C. Martins , A. D. Batista , W. R. Melchert , Compr. Rev. Food Sci. Food Saf. 2021, 20, 6116. 34564942 10.1111/1541-4337.12821

[51] a) M. Shahid , S. Manoharadas , H. Chakdar , A. F. Alrefaei , M. F. Albeshr , M. H. Almutairi , Chemosphere 2021, 278, 130372; 33839399 10.1016/j.chemosphere.2021.130372

[52] F. Zhao , L. Wang , M. Li , M. Wang , G. Liu , J. Ping , TrAC, Trends Anal. Chem. 2023, 165, 117152.

[53] Y. Wang , M. Li , Z. Wang , J. Xu , J. Zhao , Z.‐D. Gao , Y.‐Y. Song , Chem. Eng. J. 2023, 476, 146329.

[54] L. Zhang , B. Shen , C. Zheng , Y. Huang , Y. Liang , P. Fei , J. Chen , W. Lai , Food Hydrocolloids 2024, 156, 110368.

[55] L. T. Gao , Y. M. Chen , Y. Aziz , W. Wei , X. Y. Zhao , Y. He , J. Li , H. Li , H. Miyatake , Y. Ito , Carbohydr. Polym. 2024, 330, 121812. 38368083 10.1016/j.carbpol.2024.121812

[56] W. Jia , R. Fan , J. Zhang , K. Zhu , S. Gai , Y. Yin , Y. Yang , Small 2022, 18, 2201510. 10.1002/smll.202201510 35388969

