# Supplementary material for: Surface Engineering‐Induced d‐Band Center Down‐Regulation in High‐Entropy Alloy Nanowires for Enhanced Nanozyme Catalysis
Source: Adv Sci (Weinh). 2025 Apr 7;12(25):2502354. doi: 10.1002/advs.202502354 (PMC12224935; doi:10.1002/advs.202502354)
Supplement: Supplementary file 1 — Supporting Information [file ADVS-12-2502354-s001.pdf]

ADVANCED SCIENCE
Open Access

Supporting Information

for *Adv. Sci.*, DOI 10.1002/advs.202502354

Surface Engineering-Induced d-Band Center Down-Regulation in High-Entropy Alloy Nanowires for Enhanced Nanozyme Catalysis

Kunyang Feng, Hanting Wang, Song Zhou, Wei Zhang, Chonghai Gong, Yuxin He, Yusen Wang, Wenchong Dai, Jianbo Li, Zhengwei Zhang* and Siqiao Li**

Supporting Information

Surface Engineering-Induced d-Band Center Down-Regulation in High-Entropy Alloy Nanowires for Enhanced Nanozyme Catalysis

Kunyang Feng [a,b,†], *Hanting Wang* [a,b,†], *Song Zhou* [a,b], *Wei Zhang* [a,b], *Chonghai Gong* [a,b], *Yuxin He* [c], *Yusen Wang* [a,b], *Wenchong Dai* [d], *Jianbo Li* [a,b,*], *Zhengwei Zhang* [c,*], *and Siqiao Li* [a,b,*]

[a] School of Basic Medical Sciences, Chongqing Medical University, Chongqing 400016, China

[b] Chongqing Key Laboratory of Forensic Medicine, Chongqing Medical University, Chongqing 400016, China

[c] College of Pharmacy, Chongqing Medical University, Chongqing 400016, China

[d] School of Management, Shanxi Medical University, Shanxi 030001, China

*Corresponding author:

E-mail address: lijianbo@cqmu.edu.cn (J. Li); zzwcpu@cqmu.edu.cn (Z. Zhang); lsqzzw@cqmu.edu.cn (S. Li).

[†] These authors contributed equally to this work.

Experimental Section

Reagents. Ruthenium (III) acetylacetonate ($Ru(acac)_3$), platinum acetylacetonate ($Pt(acac)_2$), iron (III) acetylacetonate ($Fe(acac)_3$), nickel (II) acetylacetonate ($Ni(acac)_2$), cobalt (III) acetylacetonate ($Co(acac)_3$), oleylamine (OAm), cyltrimethylammonium bromide (CTAB), molybdenum hexacarbonyl ($Mo(CO)_6$), glucose, and acetylthiocholine chioride (ATCh) were purchased from Macklin Biochemical Technology Co., Ltd (Shanghai, China). 2,2′-azino-bis (3-ethylbenzothiazoline-6-sulfonic acid) diammonium salt (ABTS), 3,3′,5,5′-tetramethylbenzidine (TMB), and o-phenylenediamine (OPD) were obtained from Aladdin (Shanghai, China). Ethanol and H_2O_2 (30%) were obtained from Chongqing Chuandong Chemical Co., Ltd (Chongqing, China). Acetylcholinesterase (AChE) and NaAc Buffer (pH 3.5, 4.0, 4.5, 5.0, 5.5, 6.0, 6.5, and 7.0; 0.2 M) were purchased from Shanghai Yuanye Biotech Co., Ltd (Shanghai, China). Horseradish peroxidase (HRP), glucose oxidase (GOx), papain, neutral protease (NEP), and acid phosphatase (ACP) were obtained from Sigma Aldrich (USA). Bovine serum albumin (BSA) was purchased from Sangon Biotech Co., Ltd (Shanghai, China). Methomyl was purchased from LGC Labor GmbH (Augsburg, Germany). All reagents were used without further purification. All experimental aqueous solutions were prepared with distilled water.

Synthesis of FeCoNi NPs, RuFeCoNi NPs, and PtRuFeCoNi HEA NWs. PtRuFeCoNi HEA NWs were synthesized according to the previous report with a slight modification.[1] CTAB (60 mg), ($Ru(acac)_3$) (10 mg), ($Pt(acac)_2$) (8 mg), $Fe(acac)_3$ (6 mg), $Co(acac)_3$ (6 mg), $Ni(acac)_2$ (6 mg), $Mo(CO)_6$ (66 mg), and glucose (10 mg) were successively added into a 25 mL flask containing 4 mL of OAm. After the mixture was sonicated for 2 h under room temperature to obtain a homogeneous solution, the mixed solution was heated to 80 °C for 5 min and then to 220 °C for 90 min under magnetic stirring in an oil bath. The mixture was rapidly cooled to room temperature and centrifugated to collect the black products. An ethanol/cyclohexane mixture (v/v: 9:1) was used to wash the products three times. Finally, the sediments were lyophilized and dispersed in ethanol for further experiments. In addition, FeCoNi NPs and RuFeCoNi NPs were prepared under the same conditions and processes except for the different ingredients.

Synthesis of FeCoNi NPs@PDA, RuFeCoNi NPs@PDA, and PtRuFeCoNi HEA NWs@PDA. PtRuFeCoNi HEA NWs@PDA were synthesized through surface modification engineering reported by a previous method with minor modification.[2] PtRuFeCoNi HEA NWs (2 mg) were added into a 25 mL flask containing 9 mL of ethanol. After sonication for 30 min, 30 μL of dopamine (10 mg/mL) was added to 1 mL of Tris-HCl solution (1 M, pH 8.5) with vigorous stirring. The color of the solution slowly transitioned from light brown to black. After continuous stirring for 6 h, the product obtained from centrifuging the reaction solution was dissolved in ultrapure water. In addition, FeCoNi NPs@PDA and RuFeCoNi NPs@PDA were synthesized under identical conditions, with variations only in their ingredients.

Characterization of HEA NWs and HEA NWs@PDA. Transmission electron microscope (TEM) and high-resolution TEM (HRTEM) images were characterized by an FEI Tecnai G2 F20 TEM equipment (FEI, USA). Aberration-corrected scanning transmission electron microscopy (AC-STEM) and energy dispersive spectrometer (EDS) mapping images were obtained by a JEM-ARM300F STEM equipment (JEM, Japan). Powder X-ray diffraction (XRD) patterns were recorded on a D8 Advance X-ray diffractometer (Bruker, Germany). The K-Alpha X-ray photoelectron spectroscopy (XPS) spectrometer (Thermo, USA) was utilized to obtain the XPS data. The 720ES inductively coupled plasma optical emission spectrometry (ICP-OES) (Agilent, USA) was used to determine the compositions of nanomaterials. The Nicolet IS50 Fourier transform-infrared spectroscopy (FT-IR) spectrometer (Thermo, USA) was applied to measure the FT-IR data on the wavenumber range of 400–4000 cm^{-1}. Electron spin resonance (ESR) data was obtained by an electron paramagnetic resonance spectrometer (A300-10/12, Bruker, Germany). Zeta potential measurements were performed through a Zetasizer nano ZS90 (Malvern, UK). Ultraviolet–visible (UV-vis) absorption spectra were obtained with a UV-vis spectrophotometer (TU-1901, PERSEE, China). Absorbances at 652 nm for all test groups were measured using a microplate reader (Multiskan GO, Thermo, US).

The configurational entropy of HEA material was defined by the following equation:

$$\Delta S_{configuration} = -R \sum_{i=1}^{n} x_i ln x_i$$

($\Delta S_{configuration}$ >1.5 R, high-entropy; 1.0–1.5 R, middle (or medium)-entropy; < 1.0 R, low-entropy class.)

In this work, $\Delta S_{configuration}$ for HEA NWs = 1.83 R, classified as high-entropy materials.

Enzyme-Like Activities of HEA NWs, HEA NWs@PDA, and NIR-treated HEA NWs@PDA. The POD-like activities of HEA NWs and HEA NWs@PDA were assessed through TMB colorimetric assays. Specifically, TMB (8 μL, 10 mg mL^{-1}), or ABTS (8 μL, 10 mg mL^{-1}), or OPD (8 μL, 10 mg mL^{-1}), and H_2O_2 (2 μL, 30%) were added to a pH 4.5 NaAc buffer (2 mL, 0.2 M) containing 5 μg mL^{-1} of either FeCoNi NPs, RuFeCoNi NPs, HEA NWs, or HEA NWs@PDA. Absorbance at 652 nm was measured at room temperature following a 5-min reaction period. Additionally, NIR-treated HEA NWs@PDA were irradiated with an 808 nm NIR laser for 5 min. To determine the optimal conditions for the enzyme-like activity of HEA NWs@PDA, various concentrations of HEA NWs@PDA (1, 2, 3, 4, 5, and 6 μg mL^{-1}), a range of pH values (3.5 to 7.0), and power densities (0.2 to 1.2 W cm^{-2}) were evaluated.

Enzyme Kinetics Studies of HEA NWs, HEA NWs@PDA, and NIR-treated HEA NWs@PDA. Kinetic measurements were conducted in time-course mode by monitoring the absorbance changes at 652 nm. Peroxidation reactions were performed with varying concentrations of TMB (0.1 to 2.0 mM) and H_2O_2 (1.0 to 10 mM). In each experiment, either HEA NWs or HEA NWs@PDA (5 μg mL^{-1}) were added to a reaction solution containing H_2O_2 and TMB substrates in NaAc buffer solution (pH 4.5, 0.2 M, 2 mL). Additionally, NIR-treated HEA NWs@PDA were irradiated with an 808 nm NIR light for 5 min. The Michaelis-Menten constant (K_m) was determined using Lineweaver-Burk plots, employing the double reciprocal of the Michaelis-Menten equation: $v = V_{max} \times [S]/(K_m + [S])$, where v represents the initial reaction velocity, V_{max} is the maximum reaction velocity, $[S]$ is the substrate concentration, and K_m is the Michaelis constant.

Specific Activity Measurement. A series of HEA NWs and HEA NWs@PDA at various concentrations (0.5–4 μg mL^{-1}) were mixed with H_2O_2 (2 μL, 30%) and TMB (8 μL, 10 mg mL^{-1}) in 2 mL of NaAc buffer solution (pH 4.5, 0.2 M). The initial reaction rate of the solution was calculated by monitoring the absorbance change of TMB

(Δa/Δt). Additionally, NIR-treated HEA NWs@PDA were exposed to 808 nm NIR irradiation for 5 min, after which their absorbance was measured. Specific activity was determined by linear fitting of the initial reaction rate and the concentration of the nanozyme.

ESR Measurement. ESR spectroscopy was used to measure the generation of hydroxyl radicals (•OH), superoxide anions (•O_2^-), and singlet oxygen ($^1O_2$). The tests were performed in the presence of H_2O_2, DMPO, and HEA NWs or HEA NWs@PDA, with all mixtures dispersed in NaAc buffer (pH 4.5, 0.2 M). After reacting at room temperature for 10 min, the solutions were immediately analyzed by ESR. Additionally, following a 5-min irradiation of HEA NWs@PDA with an 808 nm NIR light, the same ESR measurements were conducted.

DFT calculations. Spin polarized density functional theory (DFT) calculations were conducted using the Vienna ab initio simulation package (VASP)[3] with Perdew-Burke-Ernzerhof (PBE) functional[4] and projector augmented-wave (PAW) method[5] to investigate the catalytic activity. To eliminate periodic interference of the unit cell, a vacuum of at least 15 Å was added in the Z direction. The convergence tolerances for the electronic structure iteration were set to 1.0×10^{-5} eV atom^{-1} for energy and 0.03 eV Å^{-1} for maximum force. The cut-off energy was set as 500 eV. The Monkhorst-Park grid of k-point was set as 3 × 3 × 1. The DFT-D3 method of Grimme et. al.[6] was used for the van der Waals (vdW)-dispersion energy-correction.

The elementary steps are as follows:

$$H_2O_2 + * \rightarrow H_2O_2* \quad (1)$$

$$H_2O_2* \rightarrow OH*OH* \quad (2)$$

$$OH*OH* + e^- \rightarrow OH* + OH^- \quad (3)$$

$$OH* + e^- \rightarrow OH^- \quad (4)$$

$$OH*OH* + e^- + H_2O \rightarrow O* + OH* + OH^- \quad (5)$$

$$O* + OH* + e^- \rightarrow O* + OH^- \quad (6)$$

$$O* + H_2O + e^- \rightarrow 2OH^- \quad (7)$$

where * represent the active site on the surface.

Colorimetric Detection of AChE in Solution. After incubating 5 μL of AChE at

various concentrations (0–3.3 mU mL^{-1}) with 10 μL of ATCh (10 mM) at 37 °C for 30 min, 1970 μL of NaAc buffer (pH 4.5, 0.2 M), 5 μL of HEA NWs or HEA NWs@PDA (2 mg mL^{-1}), 2 μL of H_2O_2 (30%), and 8 μL of TMB (10 mM) were added to the mixture. The absorbance spectrum was measured after reacting at 25 °C for 5 min.

Colorimetric Detection of Methomyl in Solution. After incubating 5 μL of AChE (0.55 mU/mL), 10 μL of ATCh (10 mM), and 5 μL of methomyl at various concentrations (0–1000 ng mL^{-1}) at 37 °C for 30 min, 1965 μL of NaAc buffer (pH 4.5, 0.2 M), 5 μL of HEA NWs or HEA NWs@PDA (2 mg mL^{-1}), 2 μL of H_2O_2 (30%), and 8 μL of TMB (10 mM) were added to the mixture. The absorbance spectrum was measured after reacting at 25 °C for 5 min. The same measurement was also conducted by NIR-treated HEA NWs@PDA after 5 min of 808 nm NIR irradiation.

Construction of a Portable Hydrogel Colorimetric Platform for AChE and Methomyl Detection. To prepare the hydrogel, 8 mg of sodium alginate (SA) was added to a 5 mL glass vial containing 1 mL of ultrapure water and stirred at 60 °C in a water bath for 15 min. Under continuous stirring, 20 μL of $CaCl_2$ (6 mg/mL) was added, and the mixture was stirred for an additional 15 min to achieve homogeneity. Subsequently, 6 μL of HEzymes@PDA (2 mg mL^{-1}) was added to the mixture. Then, placed in a 96-well plate mold and refrigerated for 12 h to form the Hydrogel@HEzymes@PDA.

For AChE detection, 5 μL of AChE at different concentrations (0–11 mU mL^{-1}) and 10 μL of ATCh (10 mM) were incubated at 37 °C for 30 min, then added to the Hydrogel@HEzymes@PDA in the 96-well plate. Subsequently, 2 μL of H_2O_2 (30%), and 50 μL of TMB (10 mM) were added and reacted for 5 min. Color information was captured using a smartphone camera, and ImageJ software was used to convert the image into numerical data, enabling quantitative analysis of AChE.

Similarly, for methomyl detection, 5 μL of AChE (0.55 mU/mL) and 10 μL of ATCh (10 mM) were incubated at 37 °C for 30 min, then added to the Hydrogel@HEzymes@PDA in the 96-well plate. Next, 5 μL of methomyl at different concentrations (0–14 ng mL^{-1}), 2 μL of H_2O_2 (30%), and 50 μL of TMB (10 mM) were added and reacted for 5 min. The colors were recorded using a smartphone camera, and the images were processed with ImageJ software to obtain numerical values, allowing

for the quantitative analysis of methomyl.

Methomyl detection in actual samples. The analyses of methomyl residues in pear, apple, and strawberry were done to verify the feasibility and practicability of HEzymes@PDA. The spiked recovery was used to evaluate the precision of the detection method. The samples need to be pretreated before detection, and the method is as follows. Briefly, 2 g of pear was taken in 10 mL of ethanol solution and fully oscillated for 5 min, followed by ultrasound for 2 h. Then, the supernatant was collected for subsequent experiments after centrifuged for 10 min. Subsequently, incubating with 5 μL of AChE (0.55 mU/mL), 10 μL of ATCh (10 mM), and 5 μL of methomyl at various concentrations (5, 30, and 200 ng mL^{-1}) at 37 °C for 30 min. Then, 1965 μL of supernatant of fruits, 5 μL of HEzymes@PDA (2 mg mL^{-1}), 2 μL of H_2O_2 (30%), and 8 μL of TMB (10 mM) were added to the mixture. The absorbance spectrum was measured after reacting at 25 °C for 5 min. Apple and strawberry are likewise.

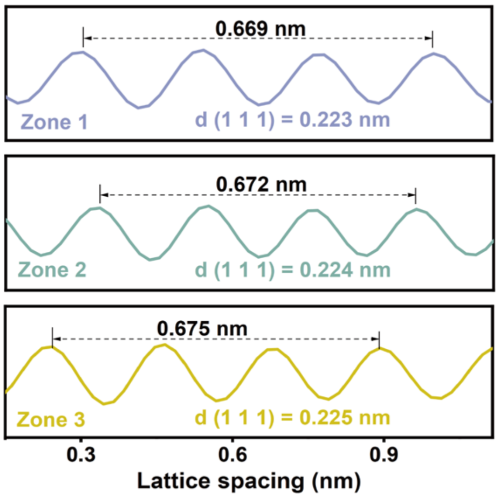

Figure S1. Integrated intensity profiles for pixels in Zone 1–3 of the HRTEM image of PtRuFeCoNi HEA NWs.

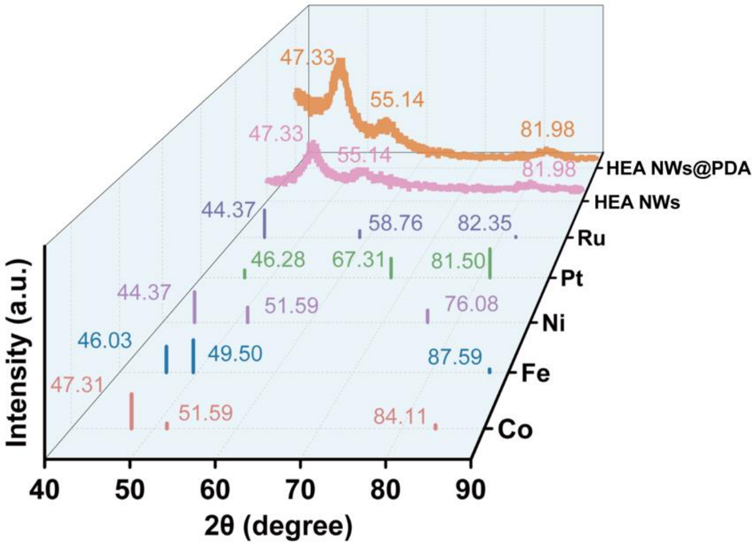

Figure S2. The XRD pattern of HEA NWs@PDA and HEA NWs versus pure metals.

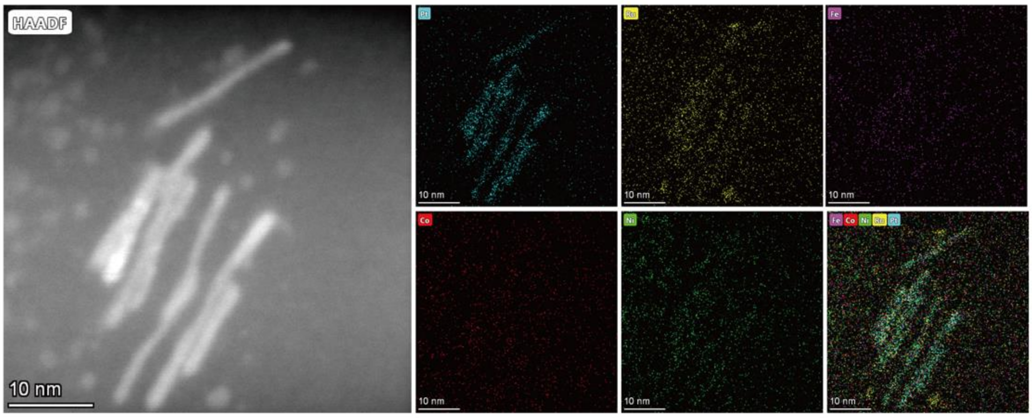

Figure S3. HAADF-STEM and corresponding elemental mapping images of PtRuFeCoNi HEA NWs.

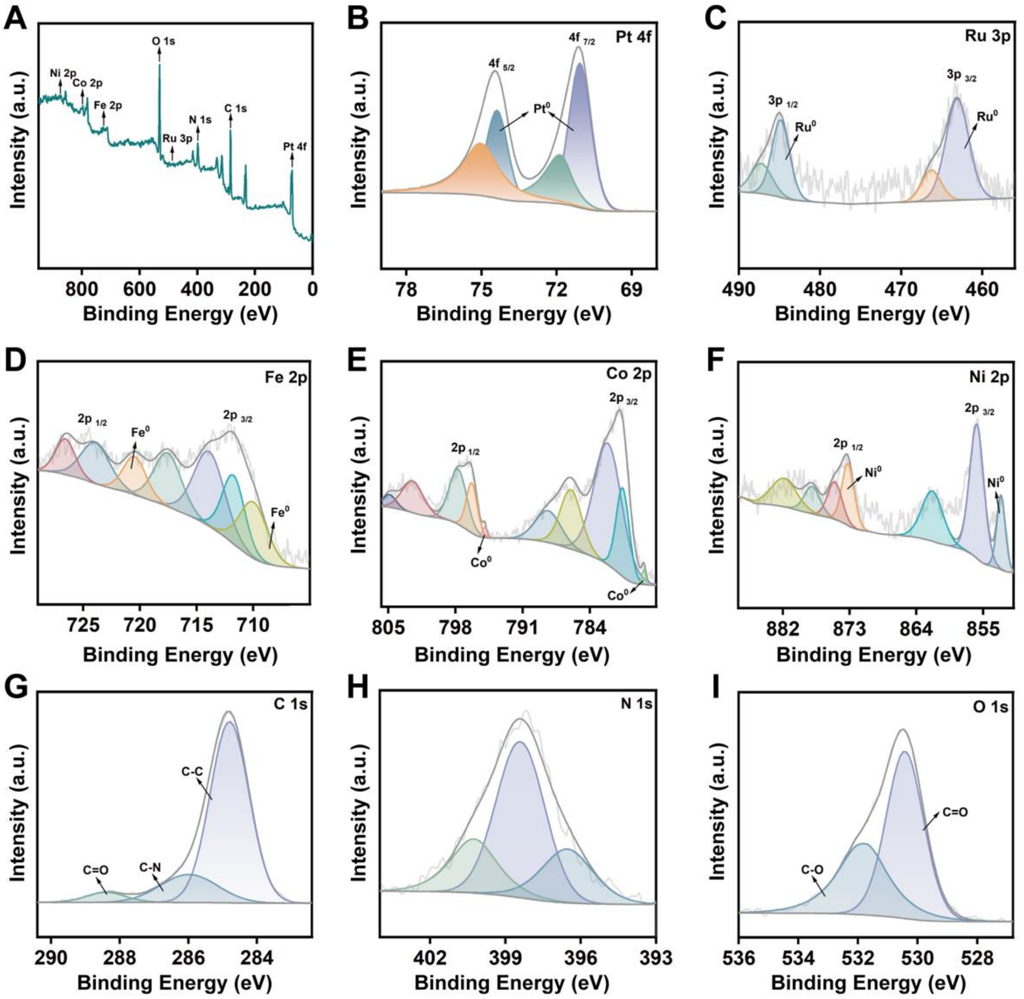

Figure S4. A) XPS spectra of HEA NWs. High-resolution XPS spectra of B) Pt 4f, C) Ru 3p, D) Fe 2p, E) Co 2p, F) Ni 2p, G) C 1s, H) N 1s, and I) O 1s for HEA NWs.

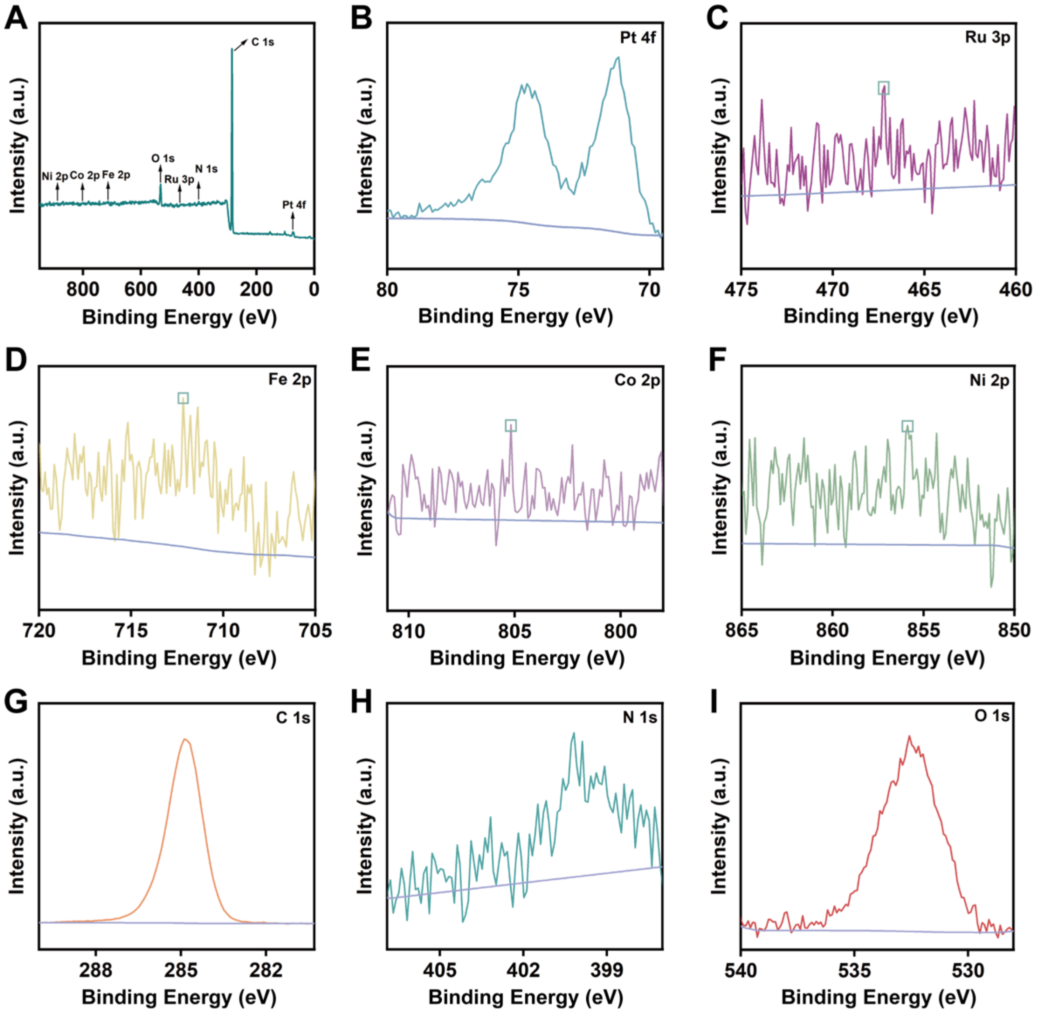

Figure S5. A) XPS spectra of HEA NWs@PDA. High-resolution XPS spectra of B) Pt 4f, C) Ru 3p, D) Fe 2p, E) Co 2p, F) Ni 2p, G) C 1s, H) N 1s, and I) O 1s for HEA NWs@PDA.

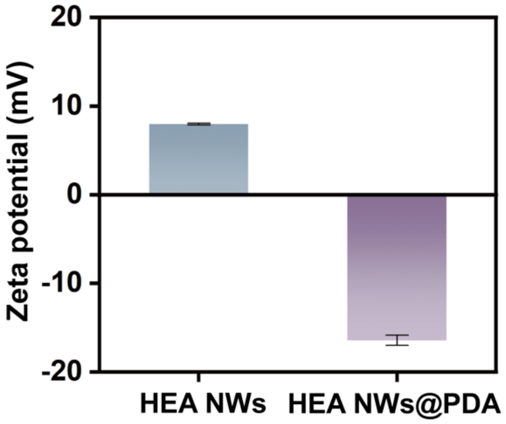

Figure S6. Zeta potential of HEA NWs and HEA NWs@PDA.

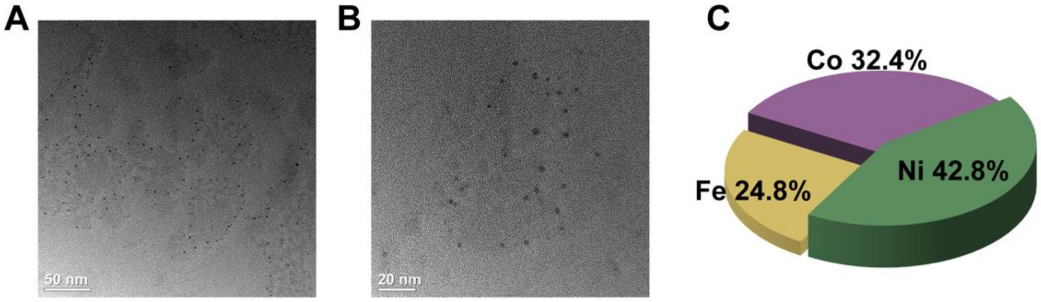

Figure S7. A,B) TEM images of FeCoNi NPs. C) Metallic element contents obtained by ICP-OES.

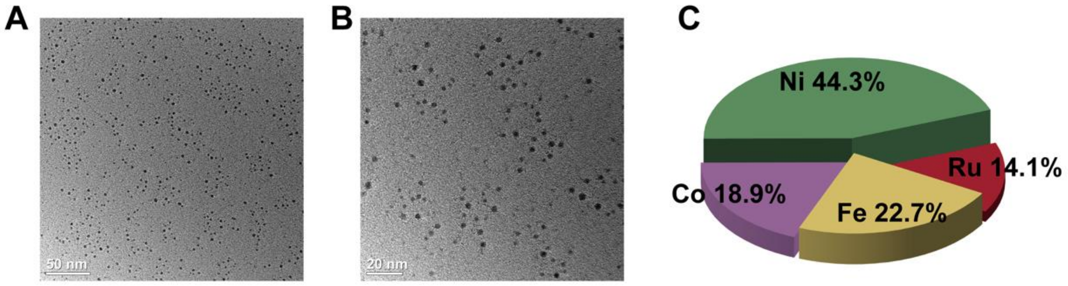

Figure S8. A,B) TEM images of RuFeCoNi NPs. C) Metallic element contents obtained by ICP-OES.

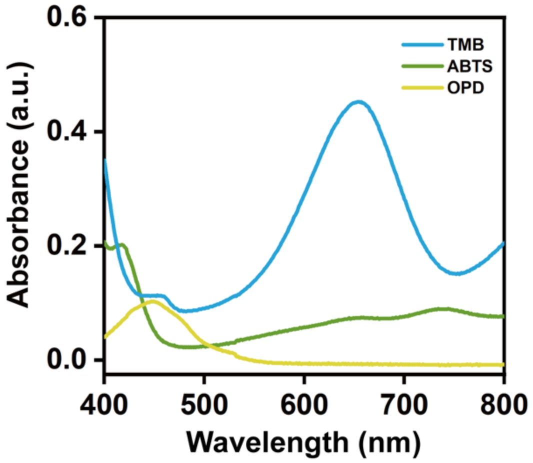

Figure S9. UV-vis absorption spectra of different chromogenic reactions of HEA NWs.

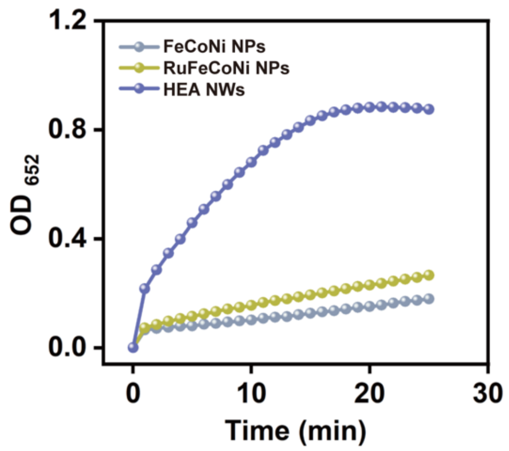

Figure S10. Time-dependent POD-like activities of FeCoNi NPs, RuFeCoNi NPs, and HEA NWs.

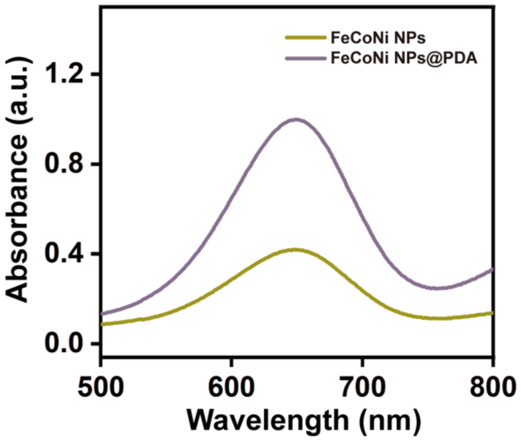

Figure S11. UV-vis absorption spectra of TMB chromogenic systems in the presence of FeCoNi NPs and FeCoNi NPs@PDA.

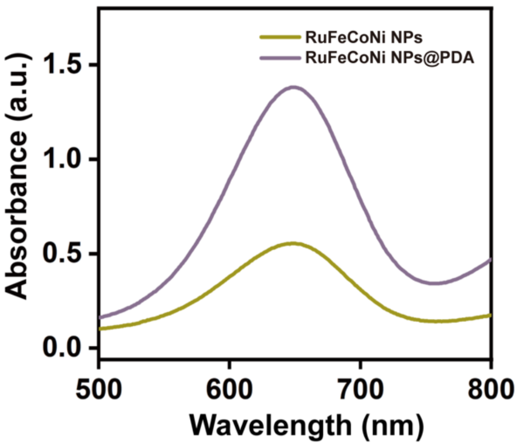

Figure S12. UV-vis absorption spectra of TMB chromogenic systems in the presence of RuFeCoNi NPs and RuFeCoNi NPs@PDA.

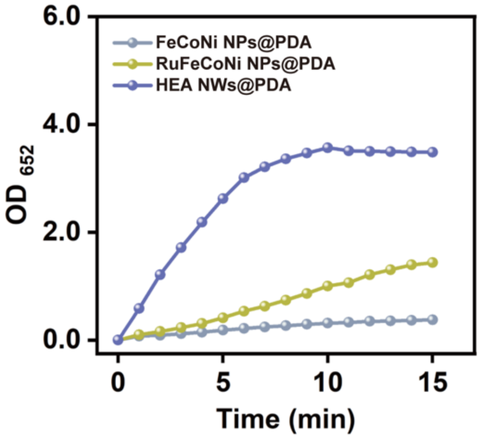

Figure S13. Time-dependent POD-like activities of FeCoNi NPs@PDA, RuFeCoNi NPs@PDA, and HEA NWs@PDA.

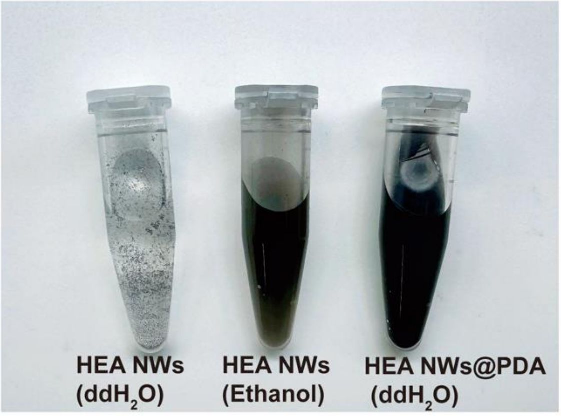

Figure S14. Optical photographs of HEA NWs and HEA NWs@PDA at room temperature with different storage solutions.

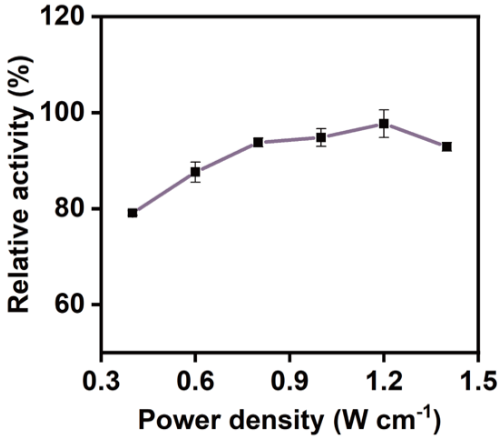

Figure S15. Effect of 808 nm laser power on POD-like activity of HEA NWs@PDA at room temperature. The data are presented as the mean ± SD (n = 3 independent samples).

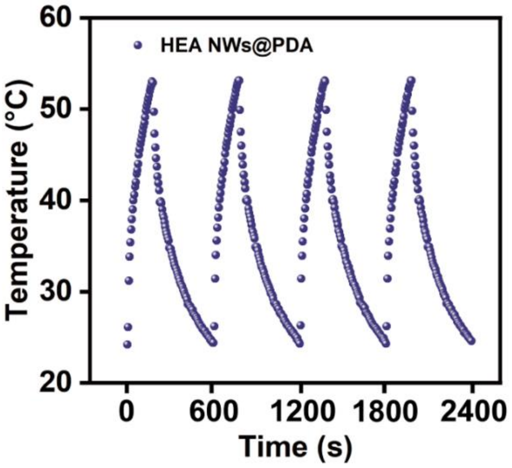

Figure S16. Photothermal stability of HEA NWs@PDA.

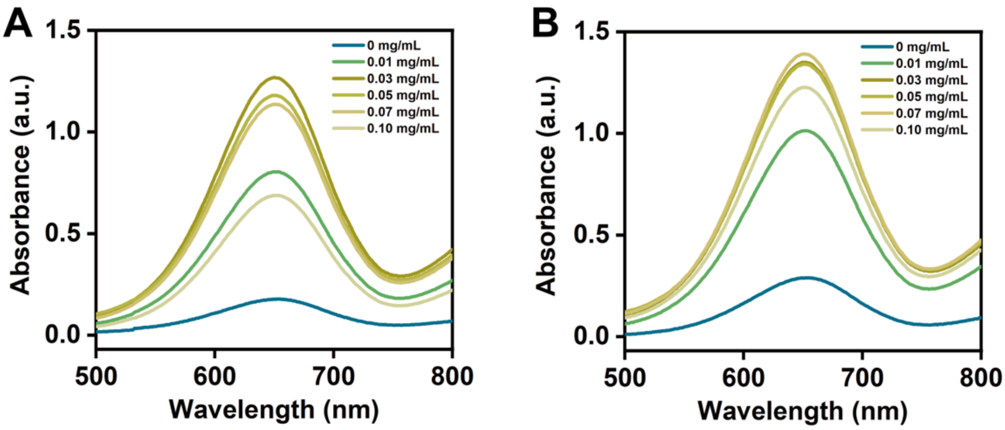

Figure S17. A) UV-vis absorption spectra of TMB chromogenic systems in the presence of HEA NWs modified with different amounts of PDA under darkness. B) UV-vis absorption spectra of TMB chromogenic systems in the presence of HEA NWs modified with different amounts of PDA under NIR light.

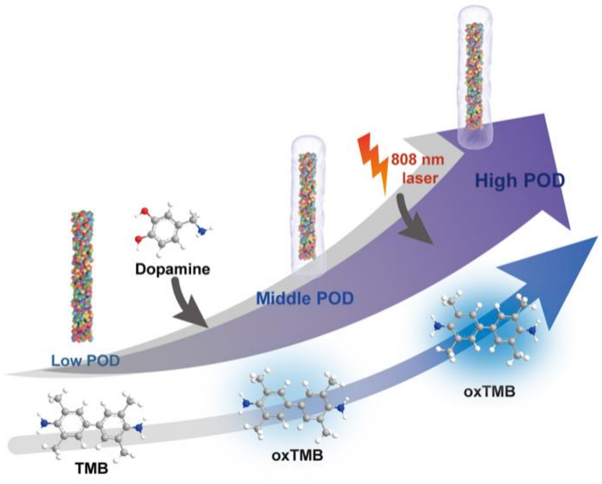

Figure S18. Schematic diagram of the two-step enhancement of POD-like activity of HEA NWs.

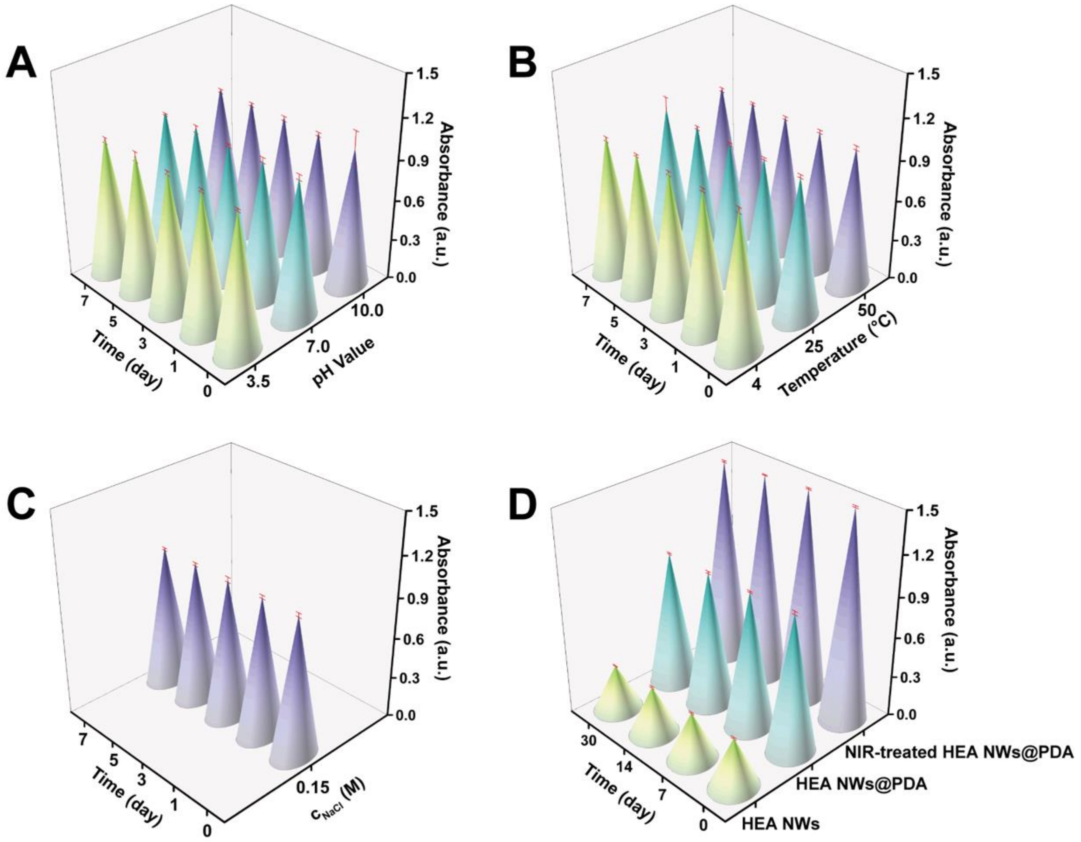

Figure S19. Stability assay of POD-like activity of HEA NWs and HEA NWs@PDA. A) Stability testing of HEA NWs@PDA at different pH values for 7 d. B) Stability testing of HEA NWs@PDA under different temperatures for 7 d. C) Stability testing of HEA NWs@PDA in 0.15 M NaCl for 7 d. D) Stability testing of HEA NWs, HEA NWs@PDA, and NIR-treated HEA NWs@PDA at room temperature for 30 d. All data are presented as mean ± SD (n = 6 independent samples).

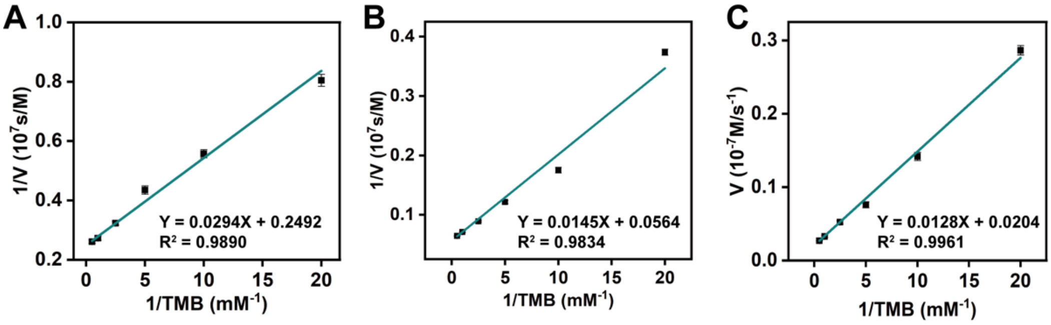

Figure S20. Lineweaver-Burk double reciprocal curve plots for A) HEA NWs, B) HEA NWs@PDA, and C) NIR-treated HEA NWs@PDA with TMB. All data are presented as mean ± SD (n = 6 independent samples).

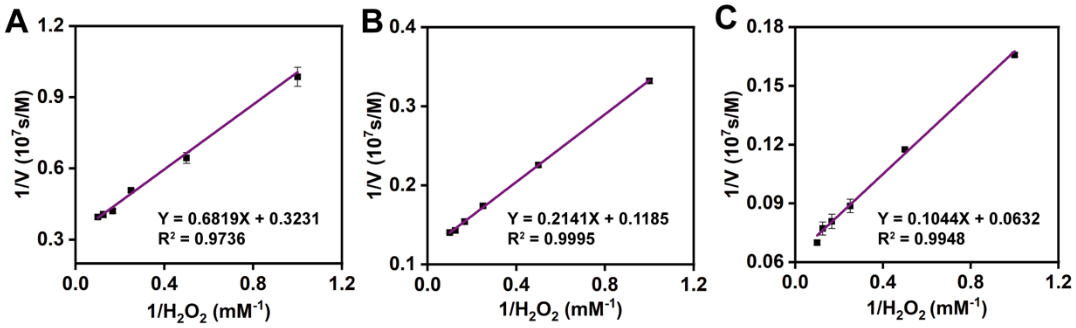

Figure S21. Lineweaver-Burk double reciprocal curve plots for A) HEA NWs, B) HEA NWs@PDA, and C) NIR-treated HEA NWs@PDA with H_2O_2. All data are presented as mean ± SD (n = 6 independent samples).

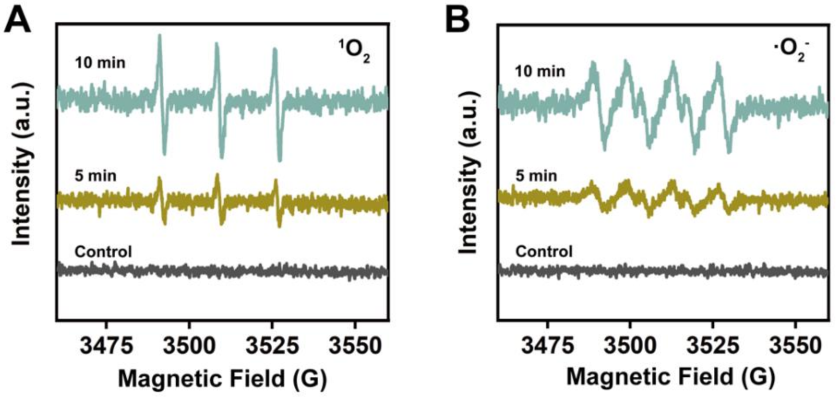

Figure S22. ESR spectra showing A) $^1O_2$ and B) $\bullet O_2^-$ formed from H_2O_2.

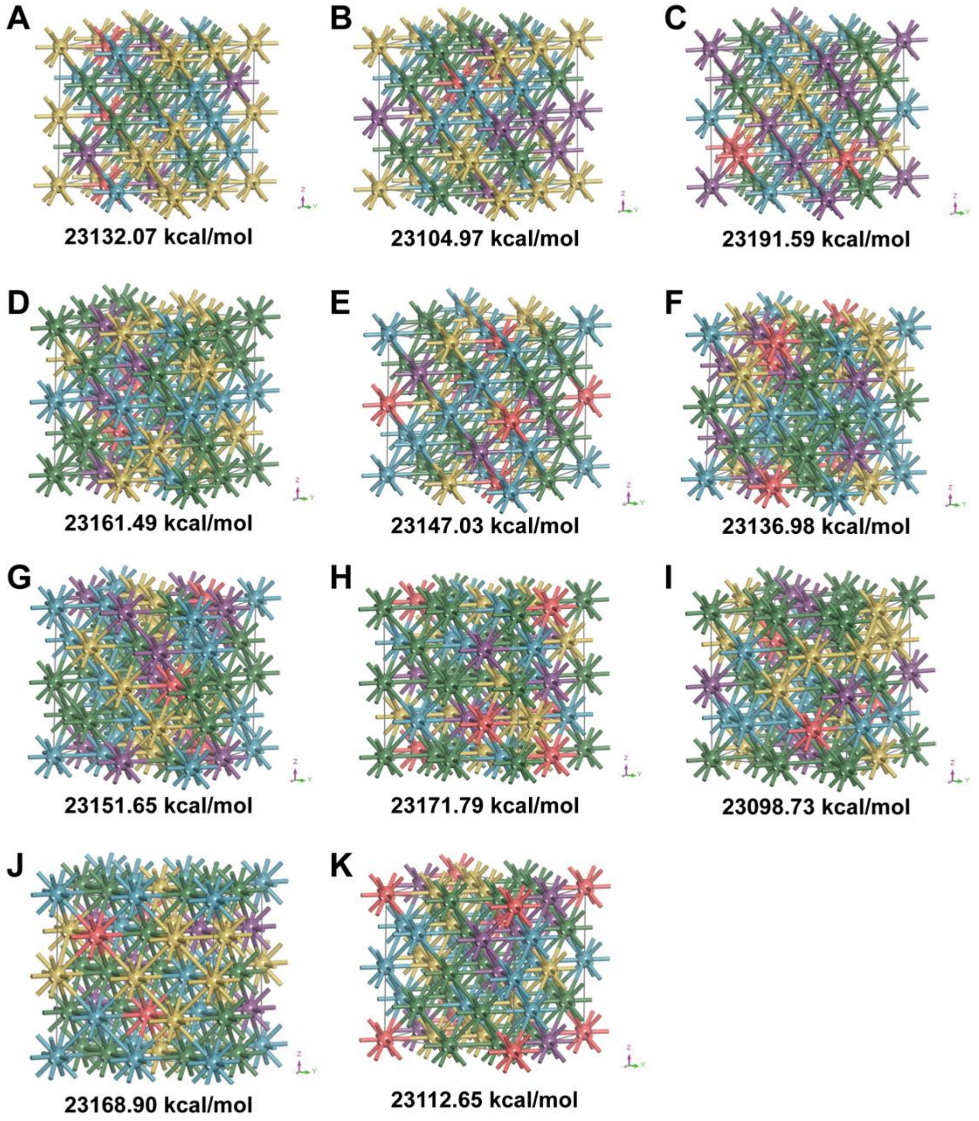

Figure S23. A–K) Structures of the optimization model for HEA NWs.

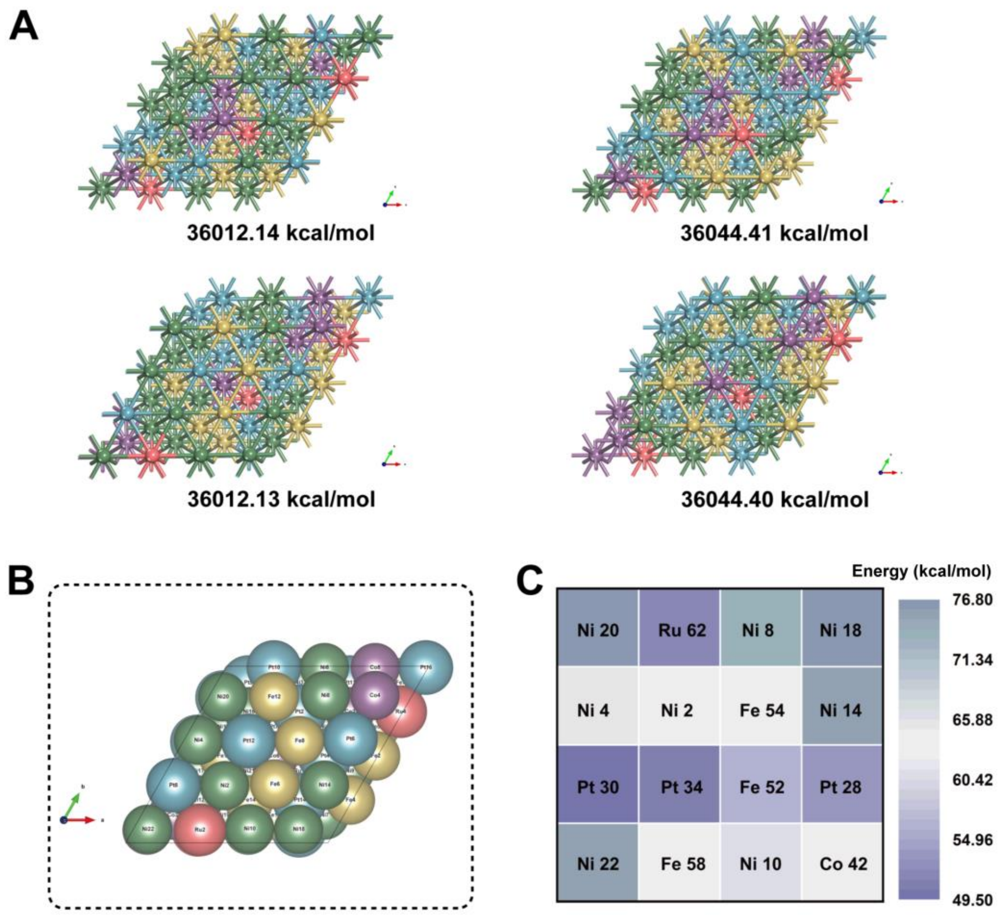

Figure S24. A) Optimization of the [111] surface energy. B) The structure of HEA NWs along the optimum [111] zone axis. C) The optimization of H_2O_2 intermediate adsorption configurations on the surface of HEA NWs.

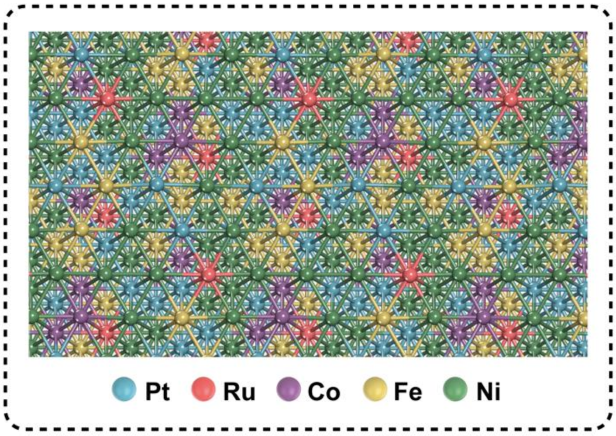

Figure S25. 3D atomic model showing the crystal structure of HEA NWs.

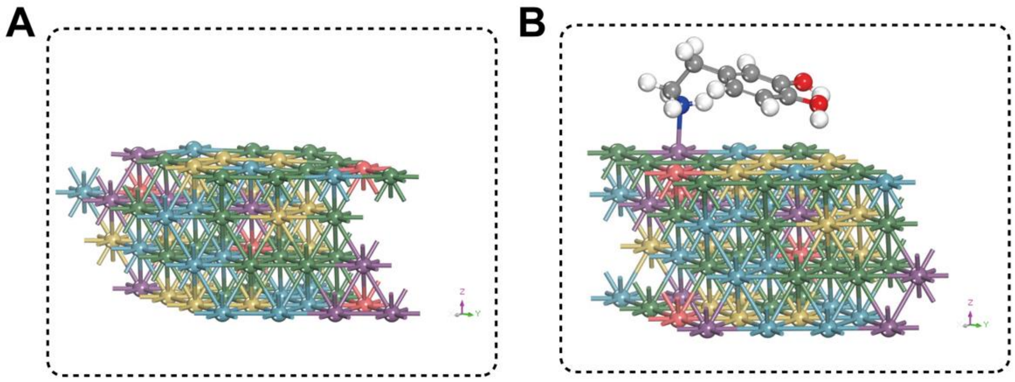

Figure S26. The optimalizing structure of A) HEA NWs and B) HEA NWs@PDA.

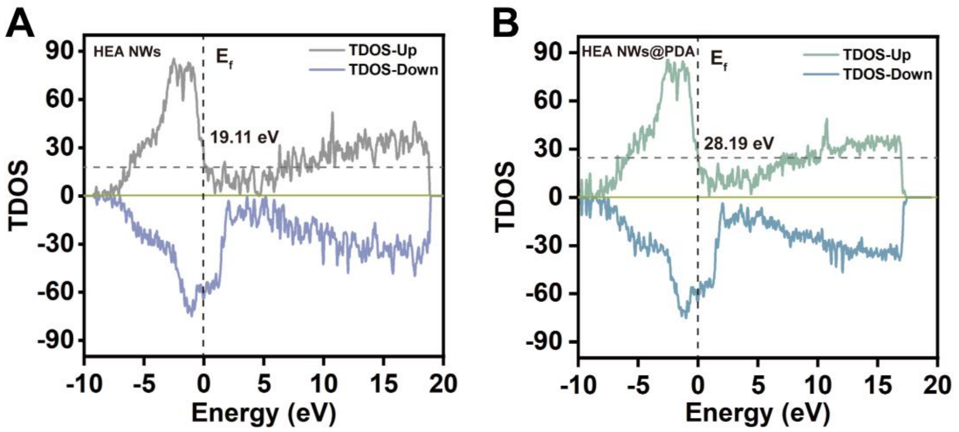

Figure S27. TDOS of A) HEA NWs and B) HEA NWs@PDA.

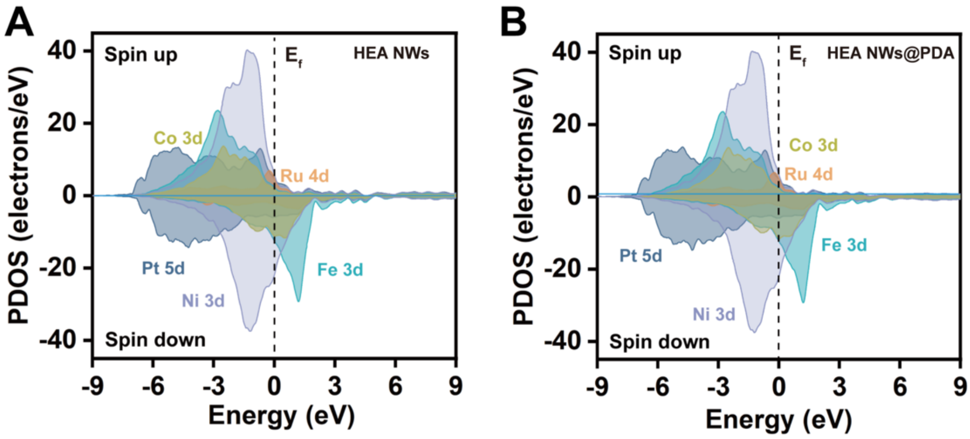

Figure S28. PDOS of A) HEA NWs and B) HEA NWs@PDA.

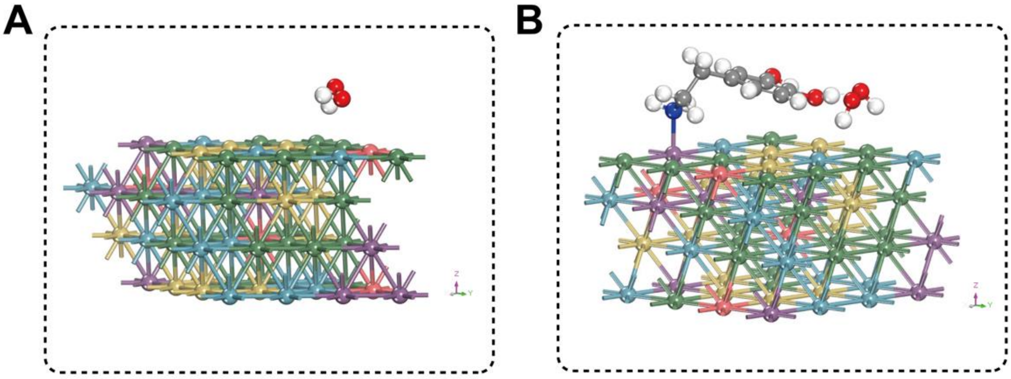

Figure S29. The optimalizing structure of H_2O_2 adsorption configurations on the surface of A) HEA NWs and B) HEA NWs@PDA.

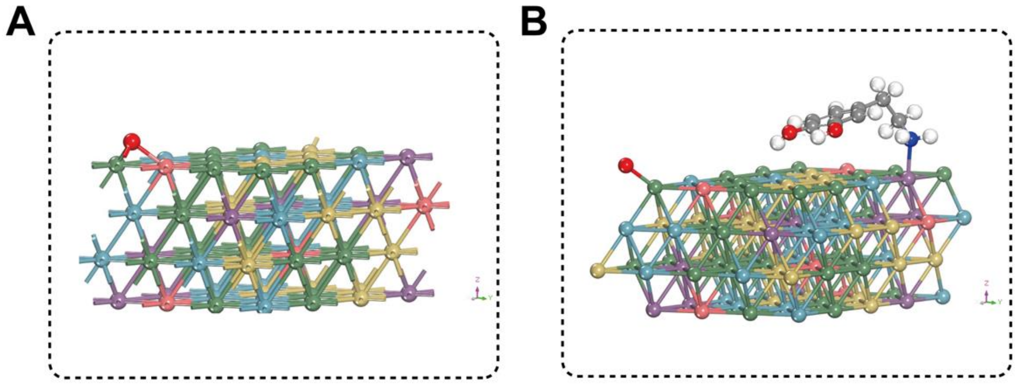

Figure S30. The optimalizing structure of oxygen-containing intermediate adsorption configurations (O*) on the surface of A) HEA NWs and B) HEA NWs@PDA.

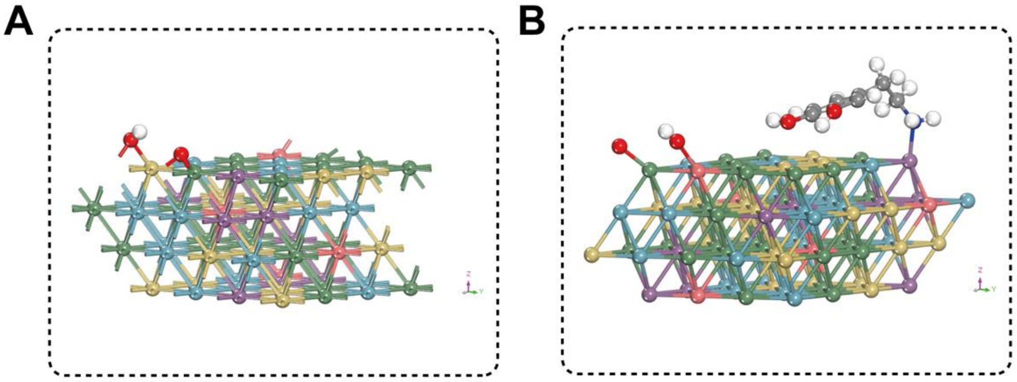

Figure S31. The optimalizing structure of oxygen-containing intermediate adsorption configurations (O* + OH*) on the surface of A) HEA NWs and B) HEA NWs@PDA.

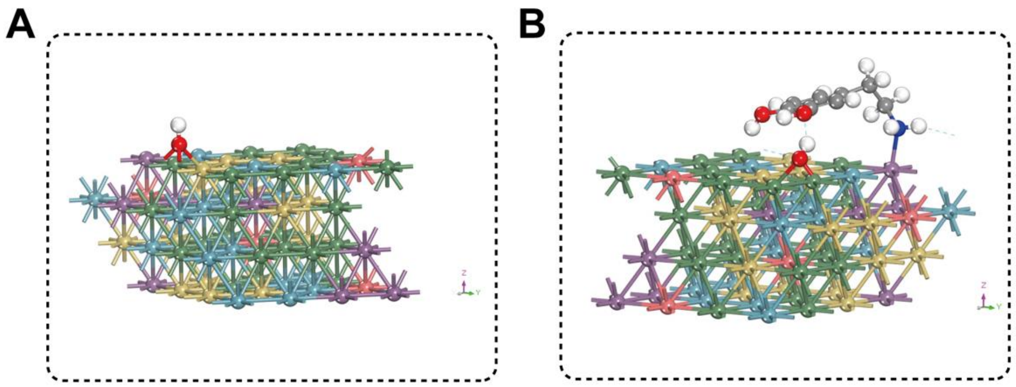

Figure S32. The optimalizing structure of oxygen-containing intermediate adsorption configurations (OH*) on the surface of A) HEA NWs and B) HEA NWs@PDA.

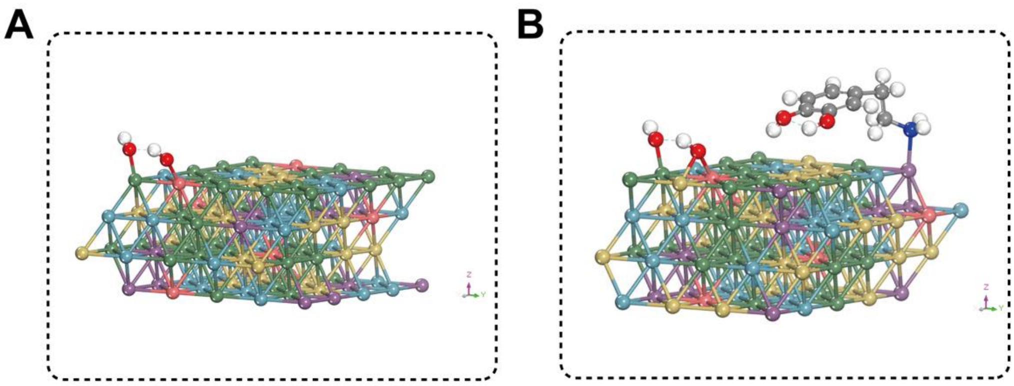

Figure S33. The optimalizing structure of oxygen-containing intermediate adsorption configurations (OH* + OH*) on the surface of A) HEA NWs and B) HEA NWs@PDA.

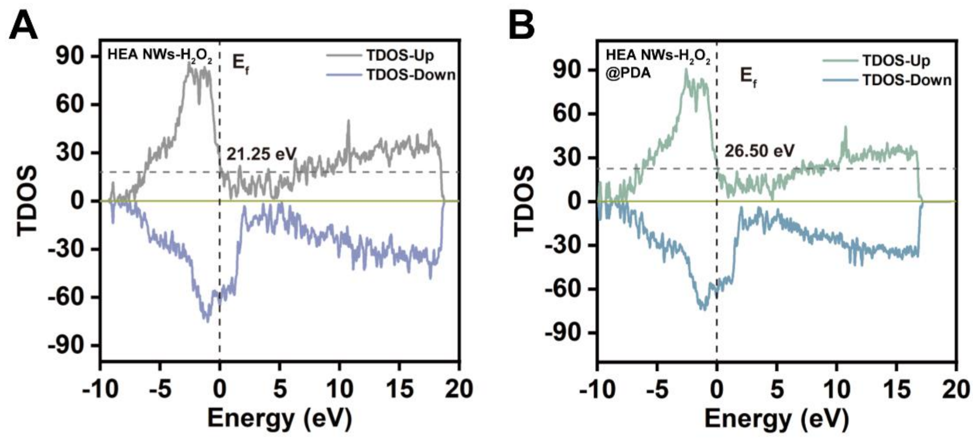

Figure S34. TDOS of for H_2O_2 adsorption on A) HEA NWs and B) HEA NWs@PDA.

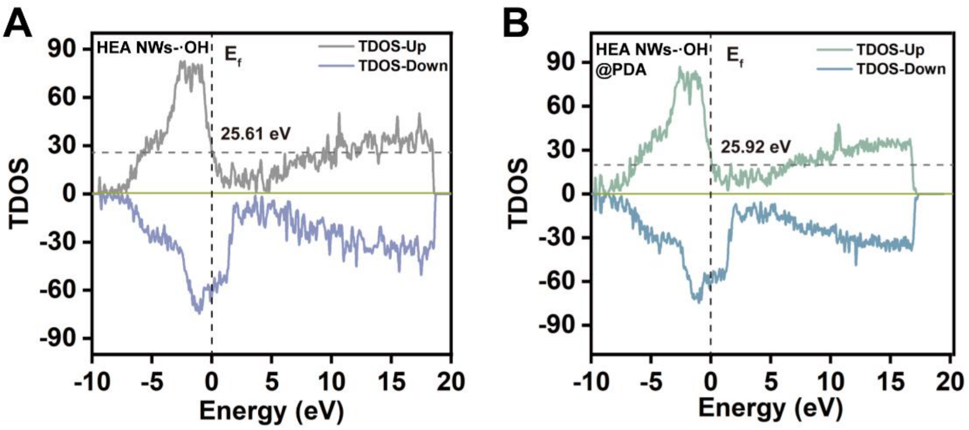

Figure S35. TDOS of for •OH adsorption on A) HEA NWs and B) HEA NWs@PDA.

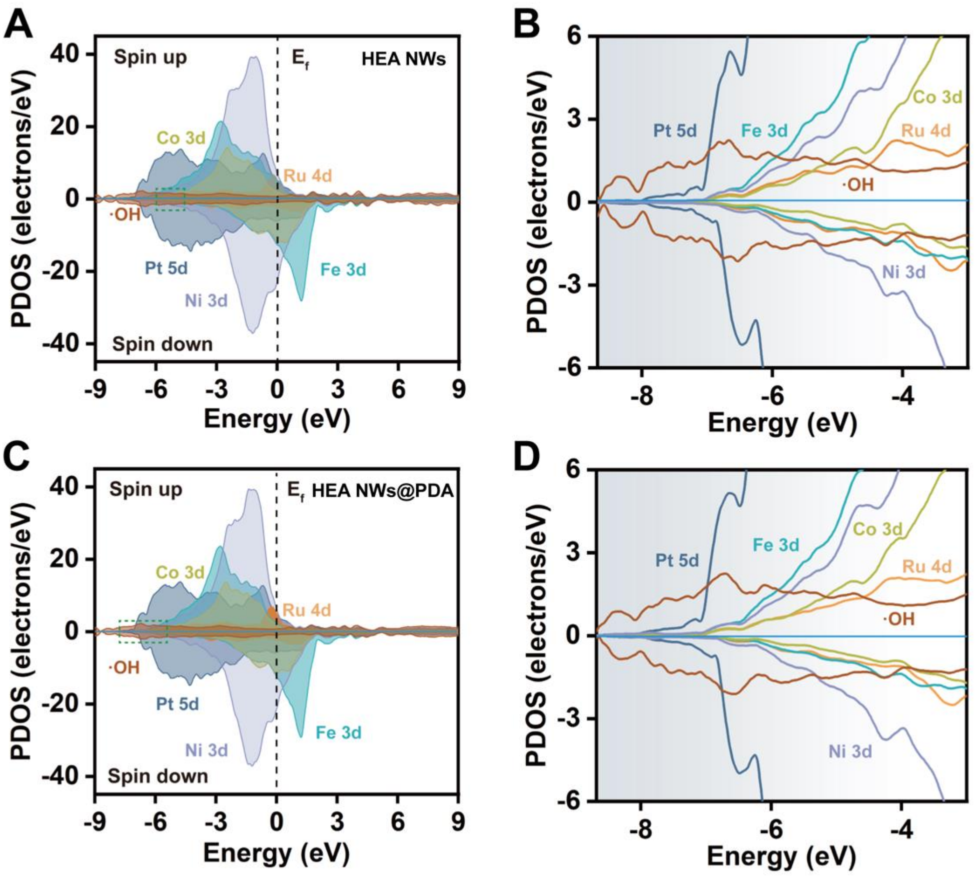

Figure S36. PDOS and partial enlargement for •OH adsorption on A,B) HEA NWs and C,D) HEA NWs@PDA.

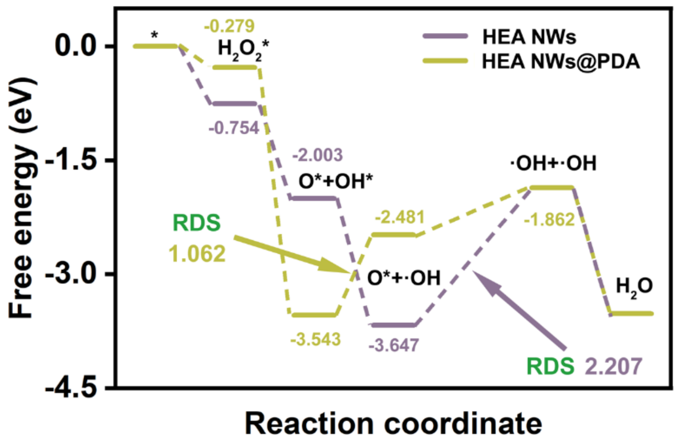

Figure S37. Optimized free energy profiles for H_2O_2 decomposition along heterolytic pathways on HEA NWs and HEA NWs@PDA.

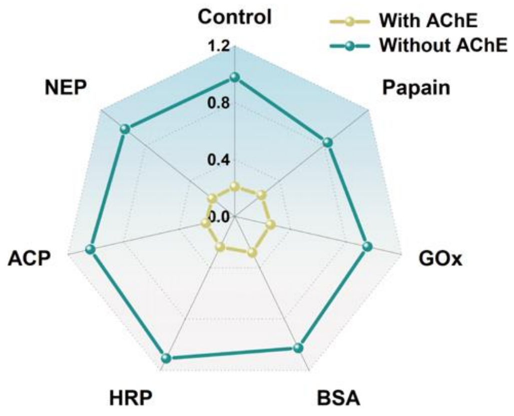

Figure S38. Interference experiment of AChE colorimetric detection platform.

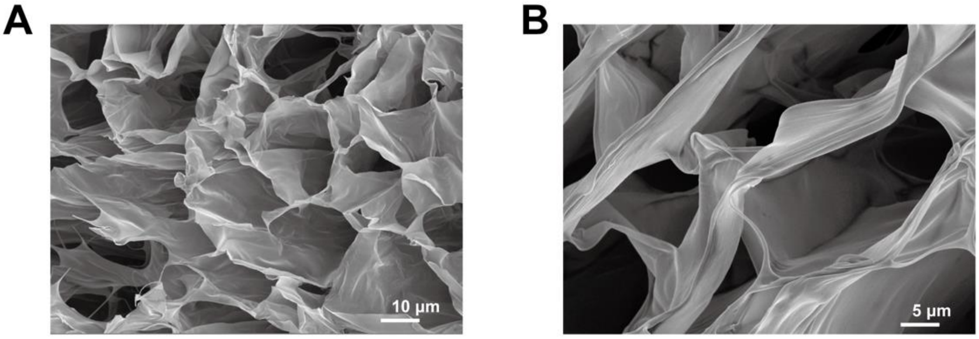

Figure S39. A,B) SEM images of SA hydrogel.

Table S1. Kinetic parameters of POD-like performance of HEA NWs, HEA NWs@PDA, and NIR-treated HEA NWs@PDA with H_2O_2.

Materials	[E] (M)	K_m (mM)	V_{max} ($\times10^{-7}$ M s^{-1})	K_{cat} (min^{-1})	K_{cat}/K_m (M^{-1} min^{-1})
HEA NWs	1.29×10^{-14}	2.11	3.09	1.44×10^{9}	6.80×10^{11}
HEA NWs@PDA	5.97×10^{-15}	1.81	8.44	8.46×10^{9}	4.67×10^{12}
NIR-treated HEA NWs@PDA	5.97×10^{-15}	1.64	15.75	1.58×10^{10}	9.60×10^{12}

Table S2. Kinetic parameters of POD-like performance of HEA NWs, HEA NWs@PDA, and NIR-treated HEA NWs@PDA with TMB.

Materials	[E] (M)	K_m (mM)	V_{max} ($\times 10^{-7}$ M s^{-1})	K_{cat} (min^{-1})	K_{cat}/K_m (M^{-1} min^{-1})
HEA NWs	1.29×10^{-14}	0.12	4.01	1.87×10^{9}	1.56×10^{13}
HEA NWs@PDA	5.97×10^{-15}	0.27	17.78	1.79×10^{10}	6.63×10^{13}
NIR-treated HEA NWs@PDA	5.97×10^{-15}	0.62	49.35	4.96×10^{10}	8.00×10^{13}

Table S3. Comparison of enzymatic kinetic parameters of HEA NWs@PDA with other nanozymes.

Catalyst	[E] (M)	Substrate	K_m (mM)	V_{max} ($\times10^{-7}$ M s^{-1})	K_{cat} (min^{-1})	K_{cat}/K_m (M^{-1} min^{-1})	Ref.
FeCoNiMnCu HEA NPs	3.09×10^{-14}	TMB	0.07	0.63	1.22×10^{8}	1.74×10^{12}	[7]
		H_2O_2	0.60	1.66	3.23×10^{8}	5.38×10^{11}	
Fe-N_4Cl/CNCl	1.92×10^{-7}	TMB	0.56	7.88	246.4	4.4×10^{5}	[8]
		H_2O_2	5.68	3.53	110.4	1.9×10^{4}	
FeN_3P-SAzyme	1.61×10^{-7}	TMB	0.002	7.75	2.89×10^{2}	1.40×10^{8}	[9]
		H_2O_2	443	5.60	3.17×10^{2}	7.16×10^{2}	
Au@CeO_2	1.67×10^{-7}	TMB	0.061	0.015	0.54	8.85×10^{3}	[10]
		H_2O_2	0.007	0.082	2.94	4.2×10^{5}	
Ir_n/ND@G	6.76×10^{-7}	TMB	1.44	11.26	99.6	6.93×10^{4}	[11]
		H_2O_2	13.49	10.66	147.6	1.09×10^{4}	
HEA NWs@PDA	5.97×10^{-15}	TMB	0.27	17.78	1.79×10^{10}	6.63×10^{13}	This work
		H_2O_2	1.81	8.44	8.46×10^{9}	4.67×10^{12}	

Table S4. Comparison of different biosensors for AChE activity assay.

Materials	Method	Linear range ($mU\ mL^{-1}$)	LOD ($mU\ mL^{-1}$)	Ref.
Fe-SAs/NC	Fluorescence	2−70	0.56	[12]
PAA-CeO_2	Fluorescence	0.263–50	0.263	[13]
Co-N-C SAzymes	Chemiluminescence	0.5–50	0.45	[14]
MOF-Pt	Chemiluminescence	0.45−15	0.40	[15]
Au@PDA NPs hydrogel	Colorimetry	2.5–25	0.9	[16]
Ag^{+}@CTAB-Au NPs	Colorimetry	0.075–25	0.075	[17]
Au-CDs	Colorimetry	0.1−5	0.107	[18]
NO_2-MIL-101	Colorimetry	0.2−50	0.14	[19]
HEzymes@PDA	Colorimetry	0.1–1.1	0.064	This work

Table S5. Comparison of different biosensors for carbamate pesticide activity screening.

Materials	Method	Linear range (ng mL^{-1})	LOD (ng mL^{-1})	Ref.
Fe_3O_4@SiO_2@$mSiO_2$	Fluorescence	52.11–997.81	50.18	[20]
AuNPs	Fluorescence	17–500	11	[21]
N-CQDs	Fluorescence	700–10000	500	[22]
PANI/CoAl-LDH	Electrochemistry	19.3–28950	1.56	[23]
Cu-CPE	Electrochemistry	13.93–2592	3.24	[24]
SACe-N-C nanozyme	Colorimetry	2×10^5–8×10^5	81.81	[25]
Ni-NPC	Colorimetry	5–100	1.5	[26]
CeO_2@NC	Colorimetry	3.81–266.39	1.256	[27]
HEzymes@PDA	Colorimetry	1–40<br>200–1000	0.329	This work
NIR-treated HEzymes@PDA	Colorimetry	0.1–5<br>100–2000	0.0628	This work

Table S6. Determination of methomyl in real samples by HEzymes@PDA.

Sample	Spiked ($ng\ mL^{-1}$)	Mean measured ($ng\ mL^{-1}$)	Mean recovery[a] (%)	RSD (%, n=3)
Pear	0	Not detected	/	/
	5.0	5.00	100.0	3.16
	30.0	30.10	100.3	2.23
	200.0	199.10	99.55	1.34
Apple	0	Not detected	/	/
	5.0	4.78	95.60	2.18
	30.0	29.39	97.97	2.87
	200.0	199.09	99.55	1.63
Strawberry	0	Not detected	/	/
	5.0	5.10	102.0	2.53
	30.0	29.95	99.83	1.81
	200.0	197.83	98.92	0.66

[a] Recovery (%) = 100 × (($C_{mean\ measured} - C_0$)/C_{spiked}).

References

[1] a) Y. Sun, W. Zhang, Q. Zhang, Y. Li, L. Gu, S. Guo, *Matter* **2023**, 6, 193; b) C. Zhan, Y. Xu, L. Bu, H. Zhu, Y. Feng, T. Yang, Y. Zhang, Z. Yang, B. Huang, Q. Shao, X. Huang, *Nature Communications* **2021**, 12, 6261.

[2] Y. Shao, W. Xu, Y. Zheng, Z. Zhu, J. Xie, X. Wei, Y. Zhang, J. Zhang, Q. Wu, J. Wang, Y. Ding, *Chemical Engineering Journal* **2023**, 455, 140586.

[3] G. Kresse, J. Furthmüller, *Computational Materials Science* **1996**, 6, 15.

[4] J. Perdew, *Physical Review Letters* **1997**, 78, 1396.

[5] P. E. Blöchl, *Physical Review B* **1994**, 50, 17953.

[6] S. Grimme, J. Antony, S. Ehrlich, H. Krieg, *The Journal of Chemical Physics* **2010**, 132, 154104.

[7] J. Feng, X. Yang, T. Du, L. Zhang, P. Zhang, J. Zhuo, L. Luo, H. Sun, Y. Han, L. Liu, *Advanced Science* **2023**, 10, 2303078.

[8] S. Wei, M. Sun, J. Huang, Z. Chen, X. Wang, L. Gao, J. Zhang, *Journal of the American Chemical Society* **2024**, 146, 33239.

[9] S. Ji, B. Jiang, H. Hao, Y. Chen, J. Dong, Y. Mao, Z. Zhang, R. Gao, W. Chen, R. Zhang, Q. Liang, H. Li, S. Liu, Y. Wang, Q. Zhang, L. Gu, D. Duan, M. Liang, D. Wang, X. Yan, Y. Li, *Nature Catalysis* **2021**, 4, 407.

[10] C. Liu, M. Zhang, H. Geng, P. Zhang, Z. Zheng, Y. Zhou, W. He, *Applied Catalysis B: Environmental* **2021**, 295, 120317.

[11] L. Yang, P. Zhu, T. Li, Y. Jiao, X. Cai, J. Liang, H. Zhang, N. Wang, X. Chen, H. Liu, *Nano Research* **2025**, 18, 94907234.

[12] M. Wang, L. Liu, X. Xie, X. Zhou, Z. Lin, X. Su, *Sensors and Actuators B: Chemical* **2020**, 313, 128023.

[13] S.-X. Zhang, S.-F. Xue, J. Deng, M. Zhang, G. Shi, T. Zhou, *Biosensors and Bioelectronics* **2016**, 85, 457.

[14] Z. Luo, L. L. Tian, H. J. Wang, Z. C. Wu, X. Luo, X. S. Wang, L. Jiao, X. Q. Wei, Y. Qin, L. R. Zheng, L. Y. Hu, W. L. Gu, L. Shi, C. Z. Zhu, *Science China-Chemisitry* **2023**, 66, 904.

[15] Y. Lu, M. Wei, C. Wang, W. Wei, Y. Liu, *Nanoscale* **2020**, 12, 4959.

[16] J. Zhang, L. Mou, X. Jiang, *Analytical Chemistry* **2018**, 90, 11423.

[17] J. Zhang, W. Zheng, X. Jiang, *Small* **2018**, 14, 1801680.

[18] D. Zhang, Q. Han, W. Liu, K. Xu, M. Shao, Y. Li, P. Du, Z. Zhang, B. Liu, L. Zhang, X. Lu, *ACS Applied Nano Materials* **2022**, 5, 1958.

[19] W. Xu, Y. Kang, L. Jiao, Y. Wu, H. Yan, J. Li, W. Gu, W. Song, C. Zhu, *Nano-Micro Letters* **2020**, 12, 184.

[20] Y. Zhu, M. Wang, X. Zhang, J. Cao, Y. She, Z. Cao, J. Wang, A. M. Abd El-Aty, *ACS Applied Nano Materials* **2022**, 5, 1327.

[21] J. Guo, Y. Luo, H. Li, X. Liu, J. Bie, M. Zhang, X. Cao, F. Shen, C. Sun, J. Liu, *Analytical Methods* **2013**, 5, 6830.

[22] S. Patel, K. Shrivas, D. Sinha, I. Karbhal, T. K. Patle, Monisha, Tikeshwari, *Spectrochimica Acta Part A: Molecular and Biomolecular Spectroscopy* **2023**, 299, 122824.

[23] W. Jiao, G. Ding, L. Wang, Y. Liu, T. Zhan, *Microchimica Acta* **2022**, 189, 78.

[24] A. Abbaci, N. Azzouz, Y. Bouznit, *Analytical Methods* **2013**, 5, 3663.

[25] G. Song, J. Zhang, H. Huang, X. Wang, X. He, Y. Luo, J.-c. Li, K. Huang, N. Cheng, *Food Chemistry* **2022**, 387, 132896.

[26] X. Xu, M. Ma, J. Gao, T. Sun, Y. Guo, D. Feng, L. Zhang, *Inorganic Chemistry* **2024**, 63, 1225.

[27] D. Zhu, N. Li, M. Zhang, Y. Wang, F. Li, T. Hou, *Biosensors and Bioelectronics* **2024**, 243, 115786.
